# Exploration of inorganic nanoparticles for revolutionary drug delivery applications: a critical review

**DOI:** 10.1186/s11671-023-03943-0

**Published:** 2023-12-19

**Authors:** Gayathri Unnikrishnan, Anjumol Joy, M. Megha, Elayaraja Kolanthai, M. Senthilkumar

**Affiliations:** 1 Department of Physics, Karunya Institute of Technology and Sciences, Coimbatore, India; 2 Department of Materials Sciences and Engineering, Advanced Materials Processing and Analysis Centre, University of Central Florida, Orlando, FL USA

**Keywords:** Inorganic nanoparticles, Drug delivery, Blood–brain-barrier, Theranostics, Wound healing

## Abstract

The nanosystems for delivering drugs which have evolved with time, are being designed for greater drug efficiency and lesser side-effects, and are also complemented by the advancement of numerous innovative materials. In comparison to the organic nanoparticles, the inorganic nanoparticles are stable, have a wide range of physicochemical, mechanical, magnetic, and optical characteristics, and also have the capability to get modified using some ligands to enrich their attraction towards the molecules at the target site, which makes them appealing for bio-imaging and drug delivery applications. One of the strong benefits of using the inorganic nanoparticles-drug conjugate is the possibility of delivering the drugs to the affected cells locally, thus reducing the side-effects like cytotoxicity, and facilitating a higher efficacy of the therapeutic drug. This review features the direct and indirect effects of such inorganic nanoparticles like gold, silver, graphene-based, hydroxyapatite, iron oxide, ZnO, and CeO_2_ nanoparticles in developing effective drug carrier systems. This article has remarked the peculiarities of these nanoparticle-based systems in pulmonary, ocular, wound healing, and antibacterial drug deliveries as well as in delivering drugs across Blood–Brain-Barrier (BBB) and acting as agents for cancer theranostics. Additionally, the article sheds light on the plausible modifications that can be carried out on the inorganic nanoparticles, from a researcher’s perspective, which could open a new pathway.

**Graphical abstract:**

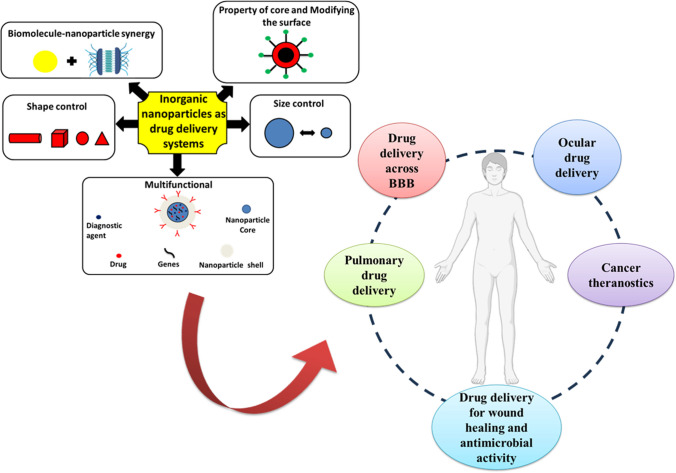

## Introduction

In earlier days, the conventional forms of drug administration were generally ointments, pills, solutions that can be injected into the bloodstream, or by oral solutions. At present, various drug delivery approaches have been developed, for example, chemical modification of drugs, entrapment of drugs in inorganic and organic materials that are placed in desired body parts, or drug entrapment in small intravenous vehicles. Drug delivery systems are those for the delivery of drugs to the target sites where therapeutic actions are to be carried out. The technologies implemented for these applications include those regarding the route of administration, drug preparation, site targeting, and toxicity. The dosage form is based on the route of administration and enteral, parenteral (injections), transdermal, inhalation, oral, and topical routes are some of the habitual routes of administration. One of the problems encountered while administering drugs is the molecular size of drug particles. Due to molecular size and charge, many medications like peptide and protein, gene, vaccine, and antibody-based drugs, may not be supplied using these routes because it cannot be absorbed into the systemic circulation effectively. The solubility of drugs also plays a vital role in drug efficacy, independent of the administration route. To avoid this problem, drug carrier materials have been used in the medical field to deliver the therapeutic molecule at specific sites in the human body. However, there are many problems still existing in drug delivery systems, such as low drug loading efficiency, rapid release, degradation rate, size, and various other surface chemistry hurdles are still some of the roadblocks and challenges after its administration to our body. There are numerous standard bulk methods to synthesize drug delivery systems, but they all suffer from several drawbacks. The constraints in generating carriers that are to be loaded with multiple therapeutic agents, studying the therapeutic/toxic effects in vivo, and the difficulty confronted in localized drug delivery can be considered as some drawbacks.

Recently, the field of nanotechnology has acquired a lot of attention for its ability to effectively diagnose and take care of various kinds of tumours. Nanocarriers are colloidal drug carrier systems having submicron particle sizes typically, less than 500 nm [1]. For the past few decades, they have been comprehensively examined as they exhibited promising results in the area of drug delivery. Nowadays, nanocarriers of size less than 100 nm have been developed. Nanocarriers have the capability of altering the fundamental properties and bioactivity of the drugs, due to their high surface area to volume ratio [2]. Nanocarrier systems own several advantageous aspects for the intended areas of applications. The volumes of distribution are lowered when the drugs and imaging agents are combined with nanocarriers. And it also can improve the pharmacokinetics and intensify the distribution of therapeutic agents to target organs, which results in better effectiveness. The drug toxicity is abridged as an outcome of its preferential accretion at the specific destinations and lower levels of concentration in the healthy tissues. Many nanoscale carriers also have the enviable advantage of enhancing the solubility of hydrophobic compounds in the aqueous medium to make them appropriate for parenteral administration. Moreover, to be on the safer side, biocompatible nanocarrier materials are being used recently [3].

The substantial amount of effort taken in the synthesis and modification of nanomaterials has resulted in the advancement of the utilization of nanoparticles for various biomedical applications such as gene/drug delivery, orthopedic implants, tissue engineering [4, 5], bone regeneration, magnetic resonance imaging (MRI), and cancer treatment [6, 7]. Nanoparticle-based drug delivery systems have arisen as an assuring methodology for the improvement of the efficiency of prevailing drugs and also to enable the advancement of new therapies. Various characteristics like size, porosity, morphology, adsorption, and physicochemical parameters determine the suitability of nanoparticles for their usage in drug delivery systems. Nanoparticles have the potential and ability to navigate through the body’s smallest blood vessels effectively and securely due to miniaturization and associated technology developments. Also, it increases the surface area for rapid dissolution of the drug. Porosity is essential for capturing gases in the nanoparticles, for the controlled release rate of the drug, and also for targeting the drugs to specified sites [8].

## Inorganic and organic nanoparticles

### Organic nanoparticles

In general, nanoparticles can be classified into two: Organic and Inorganic. Organic nanoparticles (eg: Micelles, dendrimers, ferritin, liposomes, and so on) are non-toxic and biodegradable. It also has to be highlighted that nanocapsules for example like micelles and liposomes, which have a hollow core, are delicate to thermal or electromagnetic (EM) radiations such as heat and light. These exceptional features make them a perfect choice for biomedical applications, drug delivery in particular [9]. Nevertheless, certain factors like poor stability, short shelf life and low drug encapsulation efficacy, could hinder their widespread utilization in drug delivery applications, as reported by Naseri et al. [10]. A comparison on the properties of organic and inorganic nanoparticles [11], that play a key role in determining their utilization as drug carriers, is given in Table 1.

**Table 1:** A comparison on the physical and biological properties of organic and inorganic nanoparticles

Organic Nanoparticles (eg: liposomes [12], micelles [13] etc.)	Inorganic Nanoparticles (eg: ZnO [14], Mesoporous silica [15] etc.)
Biodegradable Biocompatible Non-toxic Low stability Low reproducibility rate Issues in the drug entrapment efficiency	Ease of surface functionalization Tunable particle size Enhanced stability Improved magnetic properties Cellular toxicity Low biodegradability Less biocompatible

### Inorganic nanoparticles

Inorganic nanoparticles are highly stable and hydrophilic when compared to organic nanomaterials [16]. Inorganic nanoparticles do have intrinsic outstanding physicochemical properties (magnetic, thermal, optical, and catalytic performance) and therefore, these nanosized materials offer a sturdy framework where two or more dopants can be integrated to give multifunctional abilities [17–20]. As compared to the organic nanoparticles, the inorganic nanoparticles exhibit better drug loading capacity, excellent stability and tunable degradation rates [14, 15, 21]. Few studies based on the drug delivery systems made from inorganic nanoparticles are enlisted in Table 2 below.

**Table 2:** List of various inorganic nanoparticles loaded with various drugs

S. no	Nanoparticle used	Other materials used	Drug(s) loaded	Application	References
1	Gold nanoparticles (AuNPs)	Polyethylene-glycol (PEG)	Varlitinib	Targeted drug delivery to pancreatic cancer cells	[22]
		Chitosan/Polyacrylamide	Cisplatin	Targeted drug delivery for cancer chemotherapy	[23]
		Thioglycolic acid / Chitosan-grafted-poly(N-vinylcaprolactam)	Cisplatin	Controlled drug release	[24]
2	CaCO_3_ nanoparticles	Poly(acrylic acid) (PAA)	Doxorubicin (DOX)	pH-responsive drug delivery	[25]
		Polyethylene-glycol (PEG)	Various protein-drug combinations	Targeted co-delivery of a protein and drug	[26]
3	Mesoporous silica nanoparticles	Glutathione	Doxorubicin (DOX)	Controlled drug delivery for breast cancer cells	[27]
		Triphenylphosphine (TPP)/ Hyaluronic acid (HA)	Doxorubicin (DOX)	Targeted drug delivery system that is enzyme-responsive, and targets mitochondria and tumour cells	[28]
		Poly(amidoamine) (PAMAM)	Doxorubicin (DOX)	Drug delivery system for Bladder cancer therapy that is mucoadhesive	[29]
4	Cerium Oxide nanoparticles	Polyethylenimine (PEI)	pDNA	Gene and drug delivery to cancer cells	[30]
5	Carbon-based nanoparticles (Graphene, GO, rGO, CNTs etc.)	Polydopamine, amino- contained copolymers (Poly(PEGMA-co-NAPAM))	Doxorubicin (DOX)	Intracellular Drug delivery	[31]
		Galactosylated chitosan	Doxorubicin (DOX)	Drug delivery for tumour therapy	[32]
		Polycaprolactone (PCL), Gelatin	Bortezomib (BTZ) and Temozolomide (TMZ)	Drug delivery for glioblastoma	[33]
		Porphyrin	-	Drug delivery through blood brain barrier (BBB)	[34]
		-	Cyclophosphamide	Anticancer drug delivery	[35]
		Polystyrene	Ibuprofen	Targeted drug delivery	[36]

Inorganic nanoparticles can be generally categorized into Metal-based and Metal Oxide-based nanoparticles. The former nanoparticles are synthesized from metals and then altered to nanosize either by a bottom-up or top-down approach. Some of the familiar examples are Fe, Cu, Zn, Cd, Ag etc. The latter is synthesized to alter the properties of their corresponding metal-based nanoparticles, for example, Zinc oxide, Iron (II) oxide, Iron (III) oxide, Aluminium oxide etc. Generally, the major intention of the synthesis of these metal oxide-based nanoparticles is to increase their reactivity and efficiency. These nanoparticles hold remarkable properties when compared to their metal counterparts. In this review article, we will be discussing the recent research developments in the application of gold nanoparticles, silver nanoparticles, graphene-based nanomaterials, iron oxide, zinc oxide, hydroxyapatite and cerium oxide nanoparticles for efficient drug delivery.

#### Gold nanoparticles (AuNPs)

The properties of gold vary when they are reduced to nanoscale from the bulk form. In bulk, the colour of gold is yellowish whereas gold nanoparticles (AuNPs) exhibit various colours according to their particle size. AuNPs have attracted a remarkable amount of interest in applications ranging from photovoltaics and charge storage systems to drug delivery systems and biomolecule sensing. This is due to certain features like distinctive tunable optical and electronic properties, high X-Ray absorption coefficient, and ease of functionalization [37, 38]. We can also induce specific control on the physicochemical properties of the particle. Characteristic optical and electronic properties can be revealed by these nanoparticles with even less than 300 Au atoms, compared to bulk gold [39]. They also have a strong affinity towards amines and thiols. The electronic structure of the AuNPs tends to change with shape and size due to which, the physical properties also vary. Likewise, their chemical properties and catalytic activity are also dependent on the shape and size of the AuNPs. When the particle size is varied, the colour of the colloidal AuNPs is altered. The colour of bulk gold is yellow, but when AuNPs are of size within 2 to 100 nm, they appear red [40, 41]. This optical feature is due to the surface plasmon resonance (SPR). SPR, because of its subtle spectral response to the native environment and easiness in observing the light signal due to larger absorption and scattering cross-section, plays a key role in applications for biological sensing. The gold nanoparticles are said to have the capability of strengthening the electromagnetic field near the surface of the metal, excellent biocompatibility, high photostability, and high efficiency in light to heat conversion when compared to other chromophores.

**Fig. 1:**
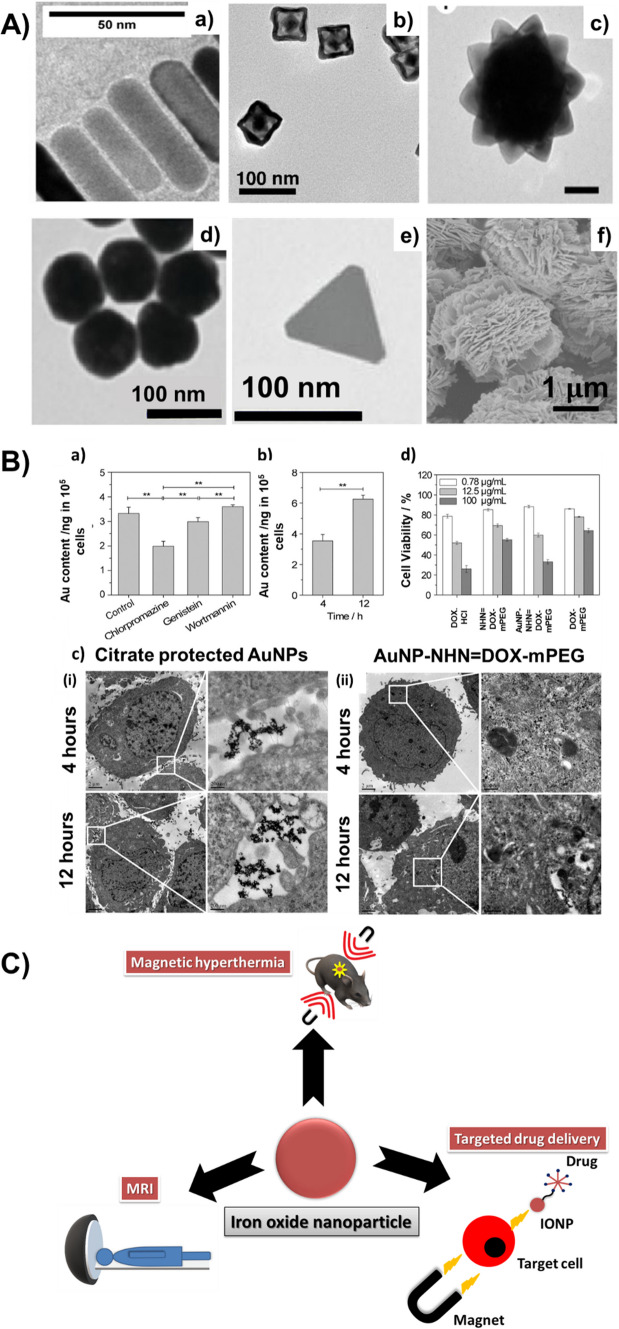
**A** TEM/SEM images of various inorganic nanoparticles having different morphologies. (a) Au nanorods, (b) Au nanorattles, (c) Au nanostars, (d) Ag nanospheres, (e) Ag nanoprisms, and (f) ZnO nanoflowers. (b–e) adapted with permission from [118, 119] and [120]. Copyright 2019, 2015, 2021 American Chemical Society. (a) and (f) adapted with permission from [121] and [122]. © 2023, 2019 Elsevier Ltd. All rights reserved. **B** The AuNPs inside the HepG2 cells treated with the AuNPs—lipoic acid—modified PEG derivative of DOX: a) in the presence and absence of various inhibitors and b) at 4 h or 12 h. c) The TEM images of HepG2 cells with: (i) citrate protected AuNPs and (ii) AuNPs—lipoic acid—modified PEG derivative of DOX for 4 h or 12 h. d) Cell viability plot. Adapted with permission from [54]. Copyright 2017 American Chemical Society. **C** Different biomedical applications of Iron Oxide nanoparticles

Generally, gold nanoparticles can be classified into three, based on dimension: 1-D (eg: nanowires, nanorods), 2-D (eg: nanoplates), and 3-D (branched structures like nanostars, and nanopods) AuNPs (Fig. 1 A (a-c)). Researchers have been investigating the anisotropic gold nanoparticles, since the early twentieth century. The properties of anisotropic gold nanoparticles were found to be different or to be more precise, exceptional when compared to that of the spherical AuNPs [42]. The anisotropy is the main reason for the plasmon absorption in both the visible and NIR region. This is why AuNPs are of great demand in diagnostics and therapies. The AuNPs can be broadly classified into two sets depending on their area of application and structure. The first set contains nanoparticles that are coupled with molecules that have different properties, and are applied for bioimaging, biosensors, targeted drug delivery, and localized hyperthermia [43, 44]. The next set contains polyfunctional, hollow AuNPs that have a gold shell and magnetic core and are commonly utilized for encapsulating the agents for therapies [45].

The functionalizations that are done to the AuNP surface bring out a drastic change in their behaviour when compared to its bulk counterpart. Thus, magnetic and optical characteristics of AuNPs are considered to be tunable by using appropriate coating because, as a result of the surface coating, there will be a change occurring in their electronic structure. The stabilization or protection offered by a shell formed from thiolate ligands, make the nanoparticles (NPs) more stable and secure towards accretion and all the other forms of decay, which facilitates attempts at recognizing different NP sizes and the assessment of how NP properties depend on size (including the quantization effects). Even though AuNPs are said to be biocompatible, their toxic effect is quite debatable. There are reports stating conclusively that the extent of AuNP toxicity highly depends on their shape, size, functionalizing material as well as the surface charge [46–50]. For instance, Au nanospheres were confirmed to be less toxic to human fetal osteoblast cells when compared to the Au nanostars and nanorods, as reported by Steckiewicz et al. But in terms of their cytotoxicity against the osteosarcoma cells, Au nanostars were reported to be most cytotoxic [51]. In another report by Wang et al., the positively charged cetyl trimethyl ammonium bromide (CTAB) coated AuNPs were reported to be highly toxic to cell membranes, in comparison to the positively charged poly (diallyl dimethyl ammonium chloride) coated AuNPs [52]. In a work by Senut et al., AuNPs of $\sim$ 1.5 nm diameter, was found toxic to the human embryonic stem cells, when compared to AuNPs of 4 nm and 14 nm diameters [53]. Therefore, special attention has to be made to the biosafety of AuNPs, while designing and synthesizing them for biomedical applications, such as drug delivery. Table 3 shows a list of very recent research developments in the area of drug delivery involving functionalized AuNPs.

**Table 3:** Recent research developments in AuNPs for drug delivery

Sl no	Material used	Highlights	Relevance	References
1	AuNPs—lipoic acid—modified PEG derivative of Doxorubicin (DOX)	-Enhanced dispersion and stability -Drug concentration in tumour cells doubled in comparison with the DOX-HCl group (Fig. 1 B)	Dual-step stimuli-responsive drug release that increases the in-vivo anti-cancer efficiency	[54]
2	AuNPs-PEG with Doxorubicin (DOX)	-Different analogs of thiolated DOX were synthesized and 2 of the most stable analogs were chosen -Release of DOX attained by the reducing agents or in the presence of an acidic environment -This reductive release was the best drug release	The resulting conjugate could be used for conjugating with cytokine TMF (tumour necrosis factor)	[55]
3	Pd-Cu@Au tripods	-Average length of tripod arm – 45.3 nm -greater action of two-photon luminescence and outstanding LSPR -Two-photon action varying with the quantity of Au present in the coating -Exceptional ability for PET imaging and targeted delivery to tumour cells	The first-ever report on a quantitative comparison of the property of two-photon luminescence of synthesized tripods to various other AuNPs	[56]
4	Ultra-small AuNPs-mesoporous silica NPs (MSNPs) with Doxorubicin (DOX)	-Redox-activated delivery of DOX -AuNPs placed in the holes of MSNPs gets heated up by NIR irradiation, which is in favour of photothermal therapy -Efficiency of photothermal conversion depended on the power of radiation and concentration of MSNP-AuNP -Faster release of DOX either by NIR irradiation or in the presence of glutathione	Drug delivery system for synergetic chemo and photothermal therapy	[57]
5	PEG-AuNPs with Bleomycin (BLM)-capped Doxorubicin (DOX)	-Exhibited greater loading capacity -Excellent stability -Displayed active targeting to cancer cells	A simple one-step synthesis of AuNPs along with conjugation of 2 anti-cancer drugs, with reduced toxicity	[58]
6	Magnetic AuNPs with Doxorubicin (DOX)	-Comprises of plasmonic Au shell -Synthesized compound exhibited high photostability -Efficient conversion of NIR light to heat energy -Increased cancer cell cytotoxicity	Multifunctional nanoplatform – magnetically targeted delivery of DOX, contrast agent of MR imaging, photothermal therapy, and chemotherapy	[59]
7	Folic acid (FA)-polymer-AuNPs with 5-fluorouracil (5FU) nanoconjugates (polymers: malate-PEG, tartrate-PEG, and citrate-PEG)	-sustained drug release up to 27 days -Highly biocompatible -Higher activities of inhibition in company with lower amounts of 5FU compared to free 5FU -π back-bonded FA-polymer-AuNP nanoconjugate	Drug delivery for breast cancer treatment	[60]
8	Doxorubicin (DOX)-PEGAuNPs and Varlitinib (Varl) -PEGAuNPs nanoconjugates	-Stable drug conjugation to PEGAuNPs (for DOX-49.5 $\pm$ 5.0%; for Varl-95.0 $\pm$ 3.0%) -Slow and stable drug release after 72 h at pH = 7.4 (47% from DOXPEGAuNPs; 31% from VarlPEGAuNPs)	Drug delivery against Human Pancreatic Adenocarcinoma	[61]
9	Extracellular Vesicles (EVs)—AuNPs/ PEG/ Folic Acid (FA)	-AuNPs are incorporated to nurture the internalization of nanoparticles and peddling of the same through the late endosome pathway, for subsequent release from cells in EVs -Enhanced uptake, the potential for immunotherapy of tumour EVs and natural tropism displayed when compared to other EVs	AuNPs helps to promote indirect labelling of EVs	[62]
10	Gold nanorods with cell-penetrating peptides (oligoarginines) and with the amphipathic peptide CLPFFD. (GNR-Arg7CLPFFD)	-Enhanced biological membrane interactions -Cell viability is least affected by the conjugate	Improvisation of cell penetration capability	[63]
11	AuNPs loaded with ketotifen	-Improved swelling of contact lens, oxygen permeability, and optical transmittance -in vitro experiments showed low burst and control ketotifen release up to 96 h	Controlled ophthalmic drug delivery	[64]
12	Au@Pt NPs—functionalised with a quinazoline based molecule	-Au NPs capped with Pt NPs -conjugation of small organic molecule (quinazoline)	Selective targeting of glioblastoma cell lines	[65]

*PEG polyethylene glycol, *Pt* platinum

#### Silver nanoparticles (AgNPs)

Silver compounds have been extensively utilized for numerous applications from utensils and jewellery to biomedical purposes. Compared to its counterpart, AgNPs hold exceptional physical, chemical, and biological properties. AgNPs are considered to have high thermal and electrical conductivities, chemical stability, and are also capable of exhibiting excellent catalytic activity and enhanced Raman scattering [66, 67]. Recent research efforts on AgNPs validated that the Ag ions possess antimicrobial, antifungal, anti-inflammatory, and antiviral properties and also have a low toxic effect on humans [68–70]. These unique properties of nanosilver paved the ground for its usage in biomedical applications including drug delivery and cancer therapy. AgNPs have a high surface area per unit mass and a stable, continuous release of Ag ions into their environment. Peculiar release profiles of Ag ions can be created by regulating the size, shape, surface coating, and agglomeration of the nanoparticles. The antimicrobial efficiency of AgNPs is attributed to the rate and duration of this release of Ag ions [71, 72]. Different sizes and shapes (spheres, wires, rods) of AgNPs can be fabricated by various synthesis methods (Fig. 1 A (d, e)). So, due to their exceptional physical and biological properties, AgNPs are being widely utilized in day-to-day life. Though, various reports explained that these AgNPs could cause adverse biological effects to both environment as well as human beings. The different factors influencing the AgNP toxicity are exogenous (eg: dosage [73]) and endogenous (eg: size [74], shape and surface functionalization [75]) factors. Table 4 is a list of research developments in the functionalized AgNPs for drug delivery applications.

**Table 4:** Recent research developments based on antimicrobial activity of AgNPs in drug delivery applications

Sl.no	Functionalization done	Effectiveness of the functionalized nanoparticle	References
1	Poly(DL-lactide-co-glycolide) (PLGA) with AgNPs	-High effectiveness against biofilm infections -Good antibacterial activity -Appropriate to build nanocarriers for biofilm infection treatments	[76]
2	Chitosan(CH)/Graphene Oxide (GO)-Ag nanocomposite hydrogel beads with Doxorubicin(DOX)	-Biocompatible and good anti-cancer effect -Improved antibacterial activity	[77]
3	AgNPs/Polyacrylamide (PAM) /Dextran (D) with Ornidazole (OD)	-Greater thermal stability, biocompatibility and also non-toxic -*in-vitro* rate of drug release—98.5% at 6 h along with 1.5% of AgNPs -Rheological study- tan $\delta$ varies from 0.1 to 0.8	[78]
4	Chitosan grafted cetyl alcohol-maleic anhydride-pyrazinamide (CS-g-(CA-MA-PZA)) with AgNPs and Rifampicin (RF)	-Higher biocompatibility and cytotoxicity effect on cells -The controlled release of RF	[79]
5	Amphotericin B, Nystatin (macrocyclic polyenes), and Fluconazole (azole) with AgNPs	-Considerable anti-amoebic properties -Better cytotoxicity	[80]
6	Fe_3_O_4_@PEG400-Ag (core/shell)	-Increased anti-bacterial (E.coli) and anti-fungal (S.aureus) activity	[81]
7	Poly(aspartic acid) -block -PCL (PAsp-b-PCL)-Ag and Doxorubicin (DOX)	Superior antitumor activity against HepG2 cells	[82]
8	Folate grafted-thiolated Chitosan (FA-TCS), Ag and Docetaxel (DTX)	DTX-Ag-NCPs (0.062 µg/ml) displayed higher anti-cancer activity in comparison with DTX-NCPs (0.536 µg/ml)	[83]
9	Pectine based Ag nanocomposite film (Pec-g- poly(AMPS-co-AAm)/Ag) with Donepezil (DPZ)	-Nontoxic and increased hemocompatibility -Release of AgNPs along with DPZ—enrichment in the activity of the drug delivery system	[84]
10	AgNPs functionalized with reduced glutathione, PEG, and Lipoic acid	-Subdued platelet growth at nontoxic concentrations -Can be used as an antiplatelet agent	[85]
11	Ag-poly-methacrylic acid (PMA) nanocapsules and Sorafenib Tosylate (SFT)	-A better release capacity of SFT-Ag-PMA capsules (about 35%) -Control over the dose, timing, and time span of the drug release when stimulated by laser irradiation	[86]
12	Chitosan microspheres (ChM)-Ag and ibuprofen	-Efficient material for inhibiting E. coli and S. aureus -Released 29.5% of loaded ibuprofen	[87]

When we look into the unique optical properties of these nanoparticles, at specific wavelengths, incident light is strongly absorbed or scattered with astonishing efficacy. This strong interaction between silver and light is because of the collective oscillation of the conduction electrons on top of the metal surface when they undergo excitation by lights of specific wavelengths i.e., a phenomenon known as Surface Plasmon Resonance (SPR). SPR is the reason for much higher absorption and scattering intensities of AgNPs when compared to similar-size non-plasmonic nanoparticles. These optical properties form the basis for the field of plasmonics and also for the analytic techniques like Surface Enhanced Raman Spectroscopy (SERS). The absorption and scattering properties are also size and shape-dependent [88]. So, they can be tuned by controlling the shape, size, and the local refractive index near the surface of the particle. When the refractive index is increased near the nanoparticle surface, the spectrum shifts towards the longer wavelength and is known as the redshift [89]. Similarly, when the refractive index is reduced near the particle surface, the spectrum shifts towards the shorter wavelength i.e., blue shift. Regarding the problems related to stability, UV–Visible spectroscopy is considered to be the most simple and dependable method to monitor the stability of AgNPs. The intensity of the original peak in the spectrum will decrease when the particles destabilize or there will be a secondary peak or a broadening of the peak, due to the accretion of particles, at longer wavelengths.

#### Graphene derivatives

Graphene has attracted huge interest from the scientific world since its first appearance. It is a single layer of sp^2^ hybridized carbon atoms organized in a honeycomb-like crystal lattice [90]. Graphene comprises of a layer of $\pi$-conjugated structure of six-atom rings, which can be theoretically regarded as a planar aromatic macromolecule. This planar structure gives it an outstanding ability to immobilize various substances like drugs, cells and other biomolecules. This is why graphene has generated much interest in the field of biomaterials and pharmaceuticals industries. Other members of the graphene family are Graphene Oxide (GO), reduced Graphene Oxide (rGO) and graphene sheets and so on. With two external surfaces, graphene has a higher surface area and it also exhibits greater biocompatibility when compared to other carbon nanoparticles like CNTs and fullerenes. In monolayer graphene, every atom in it is exposed on the surface. In comparison with other nanomaterials, this monolayer structure allows a suggestively higher drug loading capacity. The shape of graphene and GO have a significant role in the field of drug delivery. Both of them have an exceptional planar morphology and 2-D shape which is entirely different from others like tubular or spherical. If more number of layers is added to graphene, it will reduce the surface area but at the same time, increases its rigidity, which makes it perfect for cell penetration [91]. But if the structure is way too rigid, it can damage the cell. So, it is very essential to moderate the rigidity of the material to minimize the impact, which can be a hindrance for drug delivery applications. GO is easily dispersible in water and forms stable colloids whereas Graphene, on the other hand, is exceedingly hydrophobic and therefore poorly dispersible in water. It is possible to make graphene water-soluble, by adding required surfactants or by surface modification [92–97]. But the chosen synthesis method could result in several impurities, which can cause adverse biological effects [98, 99]. There are also certain reports on the toxicity of GO sheets due to their sharp edges and size. Wang et al. studied the biocompatibility of GO sheets in-vivo and observed high levels of cytotoxicity in mice after the intravenous administration of 0.4 mg GO [100]. In another study by Yuan et al., RFFCs were treated with varying concentrations of functionalized GO (GO-AgNPs). With increasing concentration of nanoparticles, the viability of cells drastically decremented, which implied a dose dependent toxicity by the particle system [73]. Thus, a balance between the therapeutic efficacy and biosafety should be maintained during the development of GO-based drug carriers. Table 5 displays the recent research developments in the area of drug delivery involving functionalized graphene-based nanoparticles.

**Table 5:** Recent applications of Graphene derivatives for various types of drug delivery

Sl. no	Functionalization	Drug	Highlights	References
1	TiO_2_@ZnO–GO	Curcumin (CUR)	-Colon targeted, pH-sensitive nanocarrier -Drug release activity was pH-dependent, due to the presence of carboxylic groups in GO -Cell viability – below 50%—anti-cancerous effect exhibited	[101]
2	Chitosan/poly(lactic acid)/GO/TiO2	Doxorubicin (DOX)	- Nanofibrous scaffolds of thickness between 30 and 50 μm -Higher sustained release rate of DOX from scaffolds in an incubation period of 2 weeks at pH 5.3 - Targeted delivery to cancer cells in the lung, in the presence of an external field	[102]
3	Folic Acid-Fe_3_O_4_@nGO	Doxorubicin (DOX)	- 50 nm-sized core–shell nanoparticles -Applicable for MR imaging due to increased magnetization saturation value -Presence of carboxyl groups due to GO coating	[103]
4	GO/Polyethylene glycol (PEG)	Doxorubicin (DOX)	-First report of GO-PEG4000 hybrid nanocarrier -Increased biodispersibility -L.E = 81%	[104]
5	GO/Polyvinylpyrrolidone (PVP)	Gefitinib (GEF) and Quercetin (QSR)	-The release profile of dual drug system was better than single drug systems -Higher cytotoxicity to PA-1 cancer cells (in the ovary), when compared to the individual drugs loaded onto the nanocomposite	[105]
6	Cobalt NPs (CoNPS)/GO/PEG	Doxorubicin (DOX)	-L.E = 196.3%, when DOX: CoNPs weight proportion is 2:1 -Capable of targeted drug delivery	[106]
7	GOMNP^*^/Polyethyleneglycol-bis-amin (PEGA)	Methotrexate (MTX)	-Lower toxicity against normal cell lines compared to free MTX -Doesn’t indicate any haemagglutination of RBCs even at high concentrations -100% release rate of the drug in 60 h, indicated more drug release in acidic conditions -Biocompatible	[107]
8	Sulfonated GO (GS)/ Chitosan (CHT)	Tetracycline Hydrochloride (TCH)	-CHT-GS exhibits continued delivery of drugs -Enhanced mechanical power when compared to CHT-GO -CHT-GO and CHT-GS show better biocompatibility	[108]
9	rGO/Chitosan (CS)	Doxorubicin (DOX)	-High biocompatibility -EE (%) = 65% -Controlled release of drug, i.e., 50% in 48 h	[109]
10	GO/ Polyethylenimine (PEI) /Au-Fe_3_O_4_	Doxorubicin (DOX) and 7-Ethyl-10-Hydroxy-Camptothecin (SN38)	- Superparamagnetic nanocomposite -Adsorption of SN38 is less than that of DOX - The release rate of DOX from nanocomposite (21% in pH 4.5 after 48 h) is better than the release rate of SN38 (15% in pH 4.5 after 48 h)	[110]
11	Fullerene (C_60_F)/ Folic acid (FA)/ chitosan (CS) /GO	*Ginkgo Biloba* Leaves polyprenol (GBP)	-GBP:C_60_F:FA:CS:GO = 100:5:4:200:200 is optimal ratio -Sustained drug release and high cytotoxicity -Low levels of genotoxicity at small concentrations of C_60_F	[111]
12	NanoGO@DOX-PEG	Doxorubicin (DOX)	-3 different molecular weights of PEG used (2 K,5 K, and 20 K) -Better cytotoxicity and increased acceptance of drugs by the cells when irradiated by NIR laser -Photothermal therapy of NGO@DOX-PEG5K was reported to be the best -pH-sensitive drug release, initiated by NIR radiation	[112]
13	Magnetic GO–NH_2_–PEG	Doxorubicin (DOX)	-non-toxic with more than 80% cellular uptake -convincing optical absorbance in the visible-NIR region	[113]
14	GO/PEG	Cephalexin (CEF)	-EE (%) = 69% -A noteworthy development in the persistent release of the drug, which can stand up to 96 h -Loading capacity—19% -Minimal adverse effects of CEF due to lower doses of the drug	[114]
15	PEG/GO/Fe_3_O_4_	Melittin (MEL)	-L.E = 370 µg/mg -numerous interactions between MEL and PEG-GO-Fe_3_O_4_ -So, the continuous and persistent release of MEL, and protected from denaturation and degradation of MEL -Higher cytotoxicity on HeLa cells -Nontoxic and biocompatible	[115]
16	GO/Chitosan (CH)/D-mannose (Ma)	Ulvan lactua	-EE (%) = 88% -A pH-dependent release behavior -Higher cytotoxicity effect against glioblastoma cells	[116]
17	GO/chitosan (CS)	Caffeic acid (CA)	- High drug loading - Release rate that did not reach zero even after 7 days	[117]

*GOMNP graphene oxide magnetic nanoparticle, *L.E.* loading efficiency, *EE* encapsulation efficiency

#### Iron oxide nanoparticles (IONPs)

Iron oxides are chemical compounds comprising of iron and oxygen. Three main and most common forms of iron oxide are Fe_3_O_4_ (Magnetite), α-Fe_2_O_3_ (Hematite), and γ-Fe_2_O_3_ (Maghemite) [123]. Greater reactive area and the capability to travel across biological barriers make the magnetic nanoparticles more favorable for drug delivery systems when compared to their microscale counterparts. Several other significant features make these magnetic nanoparticles appealing for biomedical applications (Fig. 1 C). IONPs are physically and chemically stable, biocompatible, environmentally safe, and also easily separable. At room temperature, α-Fe_2_O_3_ exhibits poor ferromagnetism but at the same time, γ-Fe_2_O_3_ and Fe_3_O_4_ are ferromagnetic. The properties of IONPs are directly related to their size and shape [124]. When IONPs are reduced to 10 to 20 nm, they build superparamagnetic properties which make them ideal for improved drug delivery systems [125, 126]. Nonetheless, the agglomeration of superparamagnetic IONPs is a common phenomenon. This can also, at times, lead to complications like toxicity. So, the biocompatibility of these nanoparticles can be enhanced by surface functionalization or suitable surface coating. It also implies that the success rate of these surface-modified IONPs based drug delivery systems will also depend on the properties of the coating material [127].

#### Zinc oxide nanoparticles

In the emerging field of nanotechnology, metal oxide nanoparticles exhibit unique physical and chemical properties owing to their limited size and a high density of corner or edge surface sites. Among the metal oxide nanoparticles, Zinc oxide (ZnO) (Fig. 1A(f)) is considered to be the most fundamental due to its distinctive physical and chemical properties, as a result of which it is implemented/exercised in many spheres. Nano ZnO enables zinc (Zn^2+^) to be easily absorbed by the body, which facilitates zinc to enact a vital role in procedures like neurogenesis and protein synthesis [128]. As we all know, nano ZnO exists in all the body tissues as an important trace element. Zinc oxide nanoparticles (ZnO NPs) are inexpensive and have less toxicity, in comparison to the other metal oxide nanoparticles. Their distinct properties include superior anti-bacterial, anti-microbial and anti-inflammatory properties [129]. The antibacterial activity may involve the accumulation of ZnO NPs in the cytoplasm of bacterial cells which trigger the release of Zn^2+^ causing bacterial cell membrane disintegration and various other activities, thereby ensuring the death of bacterial cells, although the exact mechanism remains unknown or disproven hitherto [130, 131]. Along with the aforesaid properties, ZnO NPs have high specific surface area and high activity to block a wide range of pathogenic agents, because of which they are highly efficient for many biomedical applications including drug delivery. Various drugs or biomolecules when loaded onto ZnO display better solubility, and are more effective to cancer cells, in contrast to several other individual agents. Conversely, excessive exposure to ZnO NPs results in their elevated depositions in and damage of organs such as lungs, liver, kidney, and spleen etc. irrespective of the exposure routes [132]. The generation of ROS and the oxidative stress induced by them are known to be the major toxic-mechanisms involved. Additionally, the main challenge in the application of drug delivery is in obtaining ZnO-based nanocarrier systems that are stable in-vivo. The stability requirement applies not only to ZnO nanocarriers but also to the combination of ZnO and the drugs or biomolecules to be loaded. During blood circulation, the loaded drugs should not leak and hence a strong interaction between ZnO and the load is essential. Moreover, surface modification is crucial for protecting the ZnO NPs in the biological system.

#### Cerium oxide nanoparticles or nanoceria

Cerium, a rare earth element, has two partially filled subshells (4f and 5d) and is hypothesized to have several excited substates. Cerium atom has two valence states: fully oxidized (+ 4) and fully reduced (+ 3). It has the unique property of being able to alter between the two oxidation states in redox reactions. In Cerium oxide, cerium is attached to oxygen in a crystalline fluorite lattice structure which exhibits the defects on the surface, when they are in nanoparticle form. These defects are mainly the oxygen vacancies which result in a mixed valance of Ce (IV) and Ce (III) oxidation states which coexist on the surface. This results in a redox couple which is the reason for nanoceria’s catalytic activity. This has led to an increased interest in Nanoceria as a potential biological antioxidant [133–140]. Because of the lattice structure and ease of electronic conversions with other ionic species at the quantum level, CeO_2_ NPs are capable of regenerating their redox-active matrix, allowing repetitive free radical interactions [141]. When these redox capabilities are combined with the basic properties of nanoscale particles like large surface area and quantum effects, a highly efficient nanoscale free radical scavenger can be developed [142]. CeO_2_ NPs have a cerium core surrounded by an oxygen lattice. In normal cells, under neutral pH, these NPs have an antioxidant and cytoprotective role. But as we know, one of the characteristics of the tumour cells is having an acidic nature. So, in an acidic medium, these NPs show pro-oxidant and cytotoxic effects [143]. On contrary, there are also reports stating the dose-dependent and time-dependent toxicity of CeO_2_ NPs on normal cells [144]. For example, Mittal et al. investigated the CeO_2_ NP induced toxicity in human lung cells [145]. The nanoparticles caused morphological alterations to the cells and also increased the ROS production that led to a decrement in the level of antioxidant in the cell, resulting in the cellular death. The CeO_2_ NPs are very well known for their oxygen storage capacity. When the valency of Ce changes from + 4 to + 3, oxygen is usually released into the environment. This property becomes useful in some situations while examining an intravenous activity, like the release of oxygen when the tissue oxygen is lacking [146].

#### Hydroxyapatite

Hydroxyapatite [Ca_10_(PO_4_)_6_(OH)_2_] is a calcium phosphate ceramic as well as one among the most widely utilized biomaterials, well-known for their biocompatibility and osteo-inductive properties. It is the primary mineral component found in the bones and dental tissues and therefore portrays excellent biocompatibility. Also, the nanocrystalline as well as carbonated HAp nanoparticles are regarded as biological apatite, due to the presence of OH and calcium groups [4, 5]. Aside from the dental or bone implant applications, the HAp nanoparticles are recently being utilized as drug carriers with enhanced adsorption over the biological boundaries, for controlled and targeted delivery of drugs [147–149]. Various desired drugs could be directly infused in the porous HAp or surface modified HAp nanoparticle systems, and then could be utilized for targeted delivery as well as for strengthening the nascent bone tissue structure [150–153]. These drug-loaded vehicles could be used in drug delivery applications for the bone-damage causing diseases such as osteoporosis and tumors [154–156]. Several recent reports also detailed the effective practice of using nano HAp functionalized with bioactive factors or organic polymers for targeted and controlled drug delivery, with better biodegradability, non-toxicity as well as increased drug loading capacity [157–159]. By varying the steps involved in the synthesis processes of HAp based drug carriers, like adsorption and chemical cross-linking, enhanced physical as well as mechanical characteristics could be attained [160–163]. Though, the exact dosage of the nanoparticles that is required for loading the drugs and for the achievement of desired payload release, should be optimized before the clinical trials. Therefore, the utilization of HAp nanopowder for bone-related as well as other drug delivery applications is desirable and is considered to be a biocompatible and non-toxic biopharmaceutical material. Also, the synthesis mechanism (eg: doping foreign elements or varying synthesis method) highly influences their morphological as well as other physical and biological characteristics. Thus, the regulation of physical characteristics of the nanoparticulate system is a key factor in fabricating an effective drug loading vehicle system.

## The necessity of drug delivery applications

In recent days, researchers have been more keenly concentrated on the improvement of the safety efficacy ratio of the existing drugs rather than developing a new drug that requires a huge amount of money and time. The improvisation methodologies include procedures like monitoring the drug, the drug dosage, individualization of drug therapy, slow and controlled delivery of the drug, and targeted drug delivery. The principles of pharmacodynamics and pharmacokinetics, which manage the action and character of drugs, were thoroughly studied on administering these improvised drugs on living beings. From these studies, it was found that nasal and buccal mucosa and also skin membranes can be utilized as the alternate routes for the delivery of analgesics and anaesthetics. Similarly, when these drugs were combined with other materials, a whole new range of implantable and programmable devices such as nasal aerosol sprays, transdermal and transmucosal delivery systems, incorporated with controlled drug release technology, were developed. The obstacles faced while using the standard methods of drug administration can be subjugated by implementing these methods.

In most cases, the efficiency of the drug carrier is highly dependent on the particle size, i.e., smaller the particle size, the greater the surface area and therefore has a greater capability to cross the minute parts in the human body like the blood–brain barrier and constricted junctions in the endothelial cells in the skin. The drug particles may also exhibit increased bioavailability and higher solubility. There are some basic requirements to be achieved for the designing of an ideal drug carrier for higher drug efficacy. The first and foremost one is that, when administered, the loaded drugs should reach the destination site with minimum volume loss and blood circulation activity. The next requirement is that the drug administered should only act on the desired tumour site and should be ineffective to the healthy cells.

### Types of drug delivery and the utilization of inorganic nanoparticles

#### Pulmonary drug delivery

**Fig. 2:**
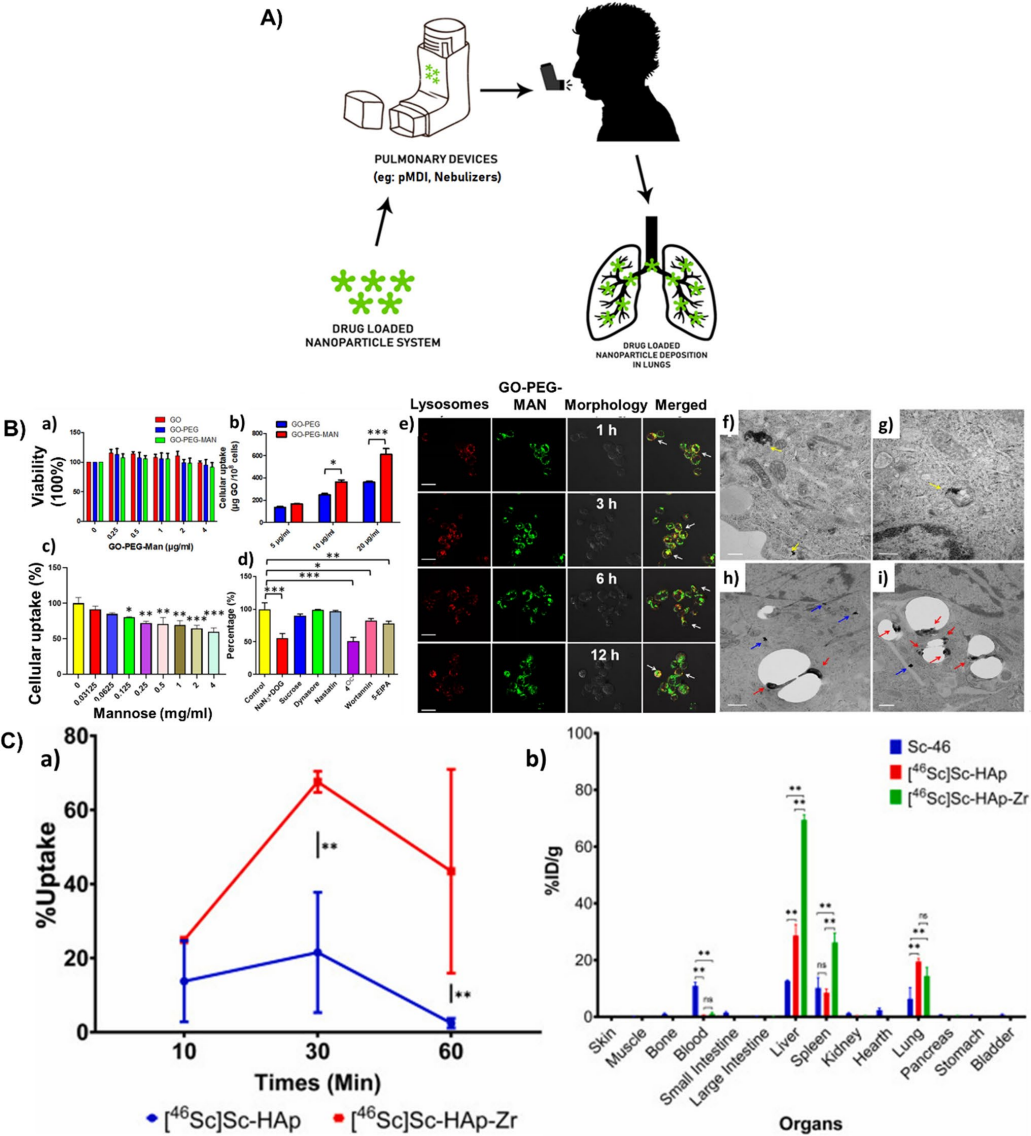
**A** Administration of drugs into lungs using nanoparticle-based drug carriers. **B** (a) Cytotoxicity of GO, GO-PEG and GO-PEG-MAN on human THP-1 cells. (b) Cellular uptake of C6@GO-PEG and C6@GO-PEG-MAN by THP-1 cells after 1 h incubation. (c) Effects of free mannose on the cellular uptake of C6@GO-PEG-MAN in THP-1 cells. (d) Cellular uptake of C6@GOPEG-MAN in THP-1 cells under different endocytosis inhibition conditions. (e) Confocal images for intracellular localization of C6@GO-PEG-MAN and lysosomes in THP-1 cells at 1 h, 3 h, 6 h and 12 h. TEM images for localization of (f, g) GO-PEG-MAN in lysosomes of THP-1 cells, and (h, i) GO-PEG-MAN and M.tuberculosis (H37Rv) in the H37Rv infected THP-1 cells. Adapted with permission from [164]. © 2019 Elsevier Ltd. All rights reserved. **C)** (a) Cellular uptake of [^46^Sc] Sc-HAp and [^46^Sc] Sc-HAp-Zr in A549 cell line at 10, 30, and 60 min. (b) Ex vivo biodistribution of Sc-46, [^46^Sc] Sc-HAp, and [^46^Sc] Sc-HAp-Zr in normal mice 1 h after injection. Adapted with permission from [165]. © 2021 Elsevier Ltd. All rights reserved

Intake of medications via inhalation has been available for many years for treating respiratory diseases. Inhalation is found to be an optimal and non-invasive route of administration for first-line therapy of asthma and other chronic obstructive pulmonary diseases. Also, lungs are capable of absorbing medications for systemic delivery, like drug delivery for diseases such as diabetes mellitus since lungs have a large surface area for absorption, high permeability, and an excellent supply of blood. The main absorption site, for most of the pharmaceuticals in the lungs, is the alveolar epithelium in the central part of the lung (Fig. 2 A). The epithelial cells in the respiratory system play a key role in regulating the airway tone and also in producing the lining fluid in the airway.

For appropriate therapeutic efficacy, if the lung is the target organ or the route of administration, a suitable amount of drug should be deposited in or around the oropharynx. In addition to the deposition site, the uniformity in the distribution of drugs is also a major factor for drug efficacy. At present, there are a number of inhaled products in the market to treat respiratory diseases. This drastic growth in the usage of inhalable drugs is due to the increased development of inhalable devices (nanoparticles or aerosols) that are capable of delivering high doses of the drug to the respiratory pathway, with higher deposition efficacy, when compared to earlier methods of administration. The capability to defeat the first-pass metabolism and low enzymatic activity are some other advantages of these newly developed inhalable devices over the traditional or peroral applications of therapeutics. When the lung is selected as the route of administration, there is a need to have some basic knowledge of the science behind the pulmonary drug delivery, i.e., physiological characteristics (respiratory pathway geometry, size of the particle, polymer selection) and other technicalities that affect the therapeutic efficiency.

There are various natural defence mechanisms like the bronchial tree, for the human lungs to prevent the entry of aerosol particles. Bronchial tree or the oropharynx are known to be very good filters that eliminate the inhaled aerosol particles and also prevent the deposition of particles in the epithelium. So, the administered drug delivery system should surpass these filters. In addition to these filters, there are some other precautionary mechanisms that inhibit the permeability of the drug into the circulatory system and cause an obstacle for the cellular uptake. It is said that, by principle, the absorption property of the substances that are inhaled greatly depends on their properties like molecular weight, charge, and solubility. Compared to the larger molecules, smaller ones are absorbed more quickly. For inhalation, the nanoparticles should possess a pH value above 3 and below 8.5, excellent aerosol properties, and should be sterile. There are few strategies followed to overcome the challenges faced during the development of inhalable therapeutic drugs using nanocarriers. The first one is the nebulization of nanocarriers in the form of colloidal suspensions. The next one is the association of the developed nanocarrier system with a microsize carrier. This association can be done by implanting the nanoparticle-based carrier into the microsize carrier. It can also be done via the incorporation of inert carriers (eg: Mannitol) onto the nanocarriers.

When we consider the case in which GO is selected as the lung targeting nanocarrier material, it was reported in previous works that the administration can be done through inhalation or IV routes and also reported the positive results based on their uptake by the cells in the lungs [166, 167]. But a detailed understanding of the chemical transformation, which occurs in the lungs after the addition of GO, is required. Liu et al. investigated the bio-transformation of GO in the lung. In their study, GO was incubated for 5 days in two lung fluids from humans, namely, Alveolar Lining Fluid (ALF) and Gamble’s solution. An increase in the C to O ratio was evident as a result of this incubation i.e., there was an increase in the number of carboxyl groups (GO-5.7%; 26% in ALF-GO and 8.7% in Gamble-GO). The interaction of GO and the loaded drug is initiated by this biotransformation. As GO, when incorporated with a poly-aromatic structure, provides an extremely larger surface area, it is very much appropriate for the effectual loading of drugs through hydrogen bonding or π-π stacking. There would be a large reduction in the surface area for the loading of drugs if the GO sheets are stacked after the transformation. From the team’s work, it was noted that the loading ability of Gamble treated GO was more constricted when compared to that of ALF treated GO since there are more carboxyl groups present on the ALF-GO surface than the Gamble-GO surface [168]. Pi et al. studied the utilization of mannosylated graphene oxide for macrophage targeted delivery of the drug, for killing intracellular *M.tuberculosis* with high efficacy. The collective release rate of Rifampicin from Rif@GO-PEG-MAN was studied at pH = 7.4 and pH = 5.5. At pH 5.5, the rate of release was found to be 27.17 ± 1.86% in 3 h, and in 48 h; it was 79.92 ± 1.72% which demonstrated an increased release rate of the drug in the acidic medium in comparison with pH 7.4 (53.62 ± 1.28% in 48 h). The study reported that the functionalization done on GO makes it an excellent material for the smart delivery of drugs (Fig. 2B) [164].

In the parenchyma of lungs, a compact system of various groups of immune cells that process and present antigens for detection by some lymphocytes (macrophages, dendritic cells, Langerhans cells), is present in addition to the large surface area of lungs. So, for treating allergic diseases, the development of nanocarriers that deliver drugs to the main immune cells for suppressing or enhancing the immune response is a very promising methodology. Fytianos et al. have investigated the immunological and functional properties and the cellular uptake of aerosolized gold nanoparticles when they are exposed to cells on the lung surface, using an innovative in vitro co-culture cell model that represented the epithelial tissue barrier in the lungs. The AuNPs were functionalized with PVA, some surface charge (PVA-NH_2_ AuNPs—positively charged and PVA-COOH AuNPs—negatively charged) and a dendritic cell-specific ICAM-3 grabbing non-integrin (DC-SIGN), which enables it to link with the receptor on the surface of dendritic cells and then labelled with a fluorescent molecule (ATTO590). They also inspected the capability of the DC-SIGN coupled gold nanoparticles in targeting the MDDCs (monocyte-derived dendritic cells), using the 3-D co-culture cell model. PVA-NH2 AuNPs, PVA-COOH AuNPs, DC-SIGN- PVA-NH2 AuNPs, and DC-SIGN- PVA-COOH AuNPs were the four different types of nanoparticles that were studied and none of them were reported cytotoxic. For all the four nanoparticles used, in general, a low occurrence of positive cells was witnessed for the epithelial cells. All the AuNPs, except DC-SIGN- PVA-NH2 AuNPs, suggestively caused slower cell death. From their results, it was evident that it is possible to develop nanoparticle-based pulmonary drug delivery systems that can be targeted to the dendritic cells and can be used to stimulate the immune reactions. They also suggested that the in-vitro system, which was developed by them, could be used for further studies like the degradation or elimination of particles or whether the particles were transported to other organs or not [169]. Gold nanoparticles were incorporated into inhalable microparticles by Silva et al. The average aerodynamic sizes of the particles used in the respirable form should be in the range between 1 and 5 $\upmu$ m so that it can reach the deep lung and supply the drug in a controlled manner, by depositing themselves on the moist surface of the lung. This study group of Silva et al. has stated that the particles, they have engineered, demonstrate a morphology that is satisfactory with an aerodynamic size which ranges from 3.2 to 3.8 $\upmu$ m. The particles exhibited a biodegradation of optimum rate. It also exhibited continuous and well-organized release of the particles loaded. As a result, there was an enhancement in the cellular uptake of particles and the authors also mentioned that their work opens a new prospect to future lung-related therapies [170].

The drug carriers, for example in the form of dry powder, are mostly administered via inhalation than any other administration routes. But the inefficiency in depositing the required amount of drugs at the desired location in the lungs is a problem that has to be rendered. Tuberculosis (TB) is known to be a fatal epidemic that claims many lives across the world each year and is extremely difficult to bring under control. Poh et al. has tried to develop a better alternative for the therapy of tuberculosis while using a respiratory treatment. They combined Bedaquiline and Q203, which are two well-known agents for treating active tuberculosis, along with SPIONs. This combination was then encapsulated into the poly (D, L-lactide-co-glycolide) and the SPIONs offer magnetic properties to the nanocarrier, therefore, it can be targeted magnetically. The particles were said to have magnetic susceptibility from the assessment done in-vitro, with magnetic saturation—28 emu/g. The efficacy in the deposition was analyzed with the help of a magnet and also by using the CFD model, and reported 100% efficient deposition in the deeper parts of the lungs [171]. Similar is the work done by Miranda et al. and the group. The group has developed a magnetically responsive microparticle system of CaCO_3_ and SPIONs, for tuberculosis treatment, where P3 is taken as the drug. SPIONs were incorporated into the microparticle system for fine targeting and controlled release of drugs from the template, with the help of an alternating external magnetic field. The developed microparticles exhibited improved and continual release of P3 at reduced pH environments. Furthermore, it was reported that the alternating external magnetic field could activate and control the rate of release of P3 since the release rate was ten times proliferated when compared to the release profile of P3 in the absence of a magnetic field. The author suggests that the inhalable drug carrier developed by them could improve the effectiveness of therapy which results in acquiescence of the patient [172]. Saifullah et al. designed a nanocarrier that comprises zinc layered hydroxide and PAS (para-aminosalicylic acid). According to the author, the developed material exhibited quadruple efficiency, in comparison with the pure PAS against *M.tuberculosis*. The inhibition concentration of the formulation developed was found to be very low (1.40 μg/mL) when compared to the minimum inhibitory concentration of pure PAS (5.0 μg/mL). It was active towards the gram-negative and gram-positive bacteria, and also against the pathogenic yeast, *Candida albicans.* The formulation was reported to be highly biocompatible and non-toxic, from in-vitro studies and also provides sustained release of PAS. The author suggested further investigation on the prepared formulation, in-vivo [173].

The capability of Nanoceria or CeO_2_ to accumulate in cells or tissues is considered to be one of its favorable properties. But, a long-time exposure and an uncontrollable concentration of CeO_2_ can cause toxicity to cells. Serebrovska et al. implanted Nanoceria onto the silica nanoparticle surface. Silica is very well known for its less toxic behaviour and also, it can be easily eradicated from living organisms. The author has reported that the CeO_2_ nanoparticles showed anti-oxidant and anti-inflammatory effects when introduced to rats. When CeO_2_ nanoparticles were introduced to both, pneumonia affected rats and normal rats, a rise in the consumption of oxygen was noted in both cases, which could be attributed to the ROS scavenging property of CeO_2_ [174]. A similar study was done by Ma et al. and has reported the ability of CeO_2_ to induce cytotoxicity and perpetual lung inflammation [175]. In the case of HAp nanoparticles, the particles with size between 100 and 200 nm, are considered as ideal for utilization as drug carriers to lungs [176]. The anticancer mechanism of these particles involves the induction of apoptosis through a mitochondria dependent method. Febrian et al. designed a Zr-doped HAp nanoparticles for cancer therapy in lungs. The particle size of the prepared Zr-HAp ranged between 119 and 138 nm. The nanoparticle system intended for lung cancer therapy, was found to have improved efficacy as the IC_50_ value was 513 $\upmu$ g/ml. The [^46^Sc] Sc-HAp-Zr nanoparticles exhibited increased accumulation in the ex-vivo lung cancer cell environment, where Sc-46 was used for radiolabelling (Fig. 2 C) [165].

#### Drug delivery for cancer theranostics

Cancer, one of the most complicated diseases, occurs due to the uncontrollable or abnormal growth of cells in different body parts and chemotherapy is the treatment where we use very strong drugs to exterminate this exceeding cell growth. The most commonly seen mode of treatment of various cancers is through the practice of toxic agents. Initially, the chemical entities used in chemotherapy interfere with the synthesis of DNA and also the process of cell division (mitosis). This will bring a halt to the division and rapid growth of cancer cells. But the drawback here is the non-selectiveness nature of therapeutic agents. But the problem arises when they act on both normal and affected cells indifferently. If the agents used, damage the normal cells along with the cancer cells, it will fail the therapy. To resolve this setback, innovative nanoparticle-based drug carriers were explored and developed in order to increase the therapeutic efficacy and also to ensure the safety of conventional cancer therapeutic agents. Many such drug carriers have been accepted, and are utilized these days. Another problematic situation is where the cancer cells pose resistance towards the drug, quite sometime after the administration. There can be two major reasons for this resistance; first, the reduction in the uptake of drugs by the cells, and second, intense leakage of drugs before reaching the desired location. Also, the acting mechanism of different drugs varies with the different kinds of cancer cells. Accordingly, the therapeutics may contain a mixture of numerous drugs. The nanoparticles have the aptitude for effective targeting and delivery of drugs due to their minimal size. They can also enhance or modify various characteristics of typical chemotherapeutic agents. So, for an intense curative effect, increased specificity, and reduced toxicity, several nanoparticle-based targeting delivery systems have been approved and developed.

Cancer theranostics is the domain which unifies the fields of diagnostics and therapy for cancer. It aims at the timely diagnosis and specific medication with appropriate dosage, along with advanced molecular imaging techniques, subsequently followed by instantaneous observation on the efficiency of treatment. For the integration of diagnostics and therapeutics, one of the best nanoparticle platforms is the gold, because of its ease in functionalization with drugs and other imaging representatives. Many researchers have assessed the capability of gold nanoparticles in delivering nuclides which are appropriate for radiotheranostics. One such study was done by Silva et al. in which AuNPs acted as a multimodal probe used for imaging purposes. They reported that the AuNPs were ornamented with BBN (Bombesin) analogues, a peptide that is bioactive and has a very high affinity to GRPr (Gastrin Releasing Peptide receptor) which are found in many kinds of tumours or cancers. They also stated that the synthesized nanoparticles were able to successfully synchronize Gadolinium (Gd^3+^) as MRI contrast agents and ^67^Ga^3+^ as SPECT imaging agents for applications in theranostics of tumours [177]. Hazkani et al. has also performed research on the modifications that can be done on AuNPs in order to make them an ideal cancer radiosensitizer and also useful for enhanced imaging. They also tried to recognize the advantages of custom-made molecular profiling for theranostics enhancement. When the tumour from the salivary gland was treated with Crizotinib-coated, anaplastic lymphoma kinase-targeted AuNPs, an enhancement in the radiative therapy was observed, which was evident from the substantial shrinkage in the volume of the tumour with time. The effect of the Crizotinib-coated AuNPs on the tumour was visualized by CT [178]. Zhao et al. developed a smart nanoparticulate probe for targeted tumour therapy and fluorescent photothermal effect, which acts only at a specific site and also can be activated reversibly. In this work, gold nanorods were functionalized with glycosyl and cyanine. Later, the particles were linked with peptides that are specific to MMP (matrix metalloproteinase) to attain reverse activation. The coated cyanine helps in giving excellent photothermal therapy when irradiated with 808 nm laser, and due to peptides linked and glycosyl, the designed probe illuminates only at the environment of the tumour. The therapy is stimulated by both pH and MMPs, and thus the devised probe is said to be dual stimuli-responsive [179]. Knights et al. studied the dependence of size and concentration of gold nanorods on its efficiency in photothermal therapy using pulsed waves and in imaging using photoacoustics. They synthesized 4 types of AuNRs of varied sizes (10, 25, 40, 50 nm), out of which, a very high photoacoustic signal was given out by large AuNRs (40 nm and 50 nm width) and high efficiency in pulsed-wave—plasmonic photothermal therapy was exhibited by the smaller AuNRs (10 nm and 25 nm). But, since the photoacoustic signal given out by nanorods of width 10 nm is also powerful enough to be detected, these nanorods were reported to be the most efficient ones (out of the 4) for both applications mentioned [180]. As far as the branched AuNPs are concerned, their number of arms and the lengths of each arm are some of the limitations for their usage in biomedical applications. Due to appealing characteristics and distinctive structure which allows the utilization of surface plasmon resonance, hollow nanostructures of gold have drawn significant interest for applications in cancer theranostics. Wang et al. prepared multifunctional, resveratrol-coated gold hollow nanoparticles through an easy and suitable procedure where no surfactants were used, which is an essential requirement in the case of theranostic tools. Resveratrol is a coating-cum-reducing agent, known for its anti-cancer and anti-oxidant activities. When irradiated using a laser ($\uplambda$ = 808 nm), the resveratrol-coated gold hollow nanoparticles efficiently inhibited the division of cancer cells and also exhibited excellent photothermal conversion efficacy [181].

Wang et al. developed a nanoprobe using Au nanostars, Atto655 (fluorescent dye), folic acid (for targeting), and DEVD (a peptide linker which is responsive to Caspase-3, an enzyme) for synchronized tumour-targeted photothermal therapy and monitoring of the imaging of Casp-3-responsive activity, which is considered as a self-therapy. When irradiated with a requisite source, the as-synthesized nanocomposite exhibited outstanding therapeutic effects by initiating the cancer cell deaths via Casp-3 pathway apoptosis, as a result of the photothermal activity. Simultaneously, the released Casp-3 concluded the process of quenching and recovered the fluorescent near-infrared signal, which helped in precisely tracing the apoptosis process [182]. Maniglio et al. developed a superparamagnetic, hydrophilic, hybrid theranostic tool, to overcome the limitations faced in theranostics like the inability in evaluating the efficacy of targeted drug delivery, from a combination of AuNPs and Fe_3_O_4_ (65:35 ratio), where Tween20 was used as a surfactant. Even at increased levels of nanoparticle concentrations, negligible cytotoxicity was exhibited. When irradiated using X-Rays, the synthesized nanoparticles persuaded the activity of radiosensitizing only selectively on osteogenic sarcoma-derived cells [183]. Srinivasan et al. designed PEG-coated AuNPs, loaded with Cyanine-7 modified with methoxyamine. The load taken is a probe that can attach to the AP sites and restrain the process of repairing DNA, which will, in turn, kill the cell. The synthesized nanoparticulate system at a ratio of 10:1 (load:AuNPs) exhibited an enhanced profile of drug release, tumour-specific confinement, and very solid attachment to the AP sites [184]. Grabowska-Jadach et al. developed a biocompatible nanoplatform that is capable of performing drug delivery and photothermal therapy for skin tumours, with hollow Au nanoshells synthesized by reducing Tetrachloroauric acid onto AgNP templates, and then conjugating it with AS1411, an aptamer with a thiol group at its end. This conjugation helped in improving the confinement of nanoparticles to the target cells than to the normal cell lines. When irradiated using a laser, the synthesized nanosystem was utilized for the production of heat and ablating the tumour cells due to the adjustable plasmonic characteristics of the AuNPs. At high concentration levels, a loss of < 10% alone was caused by the nanoconjugate in the case of cell viability [185].

**Fig. 3:**
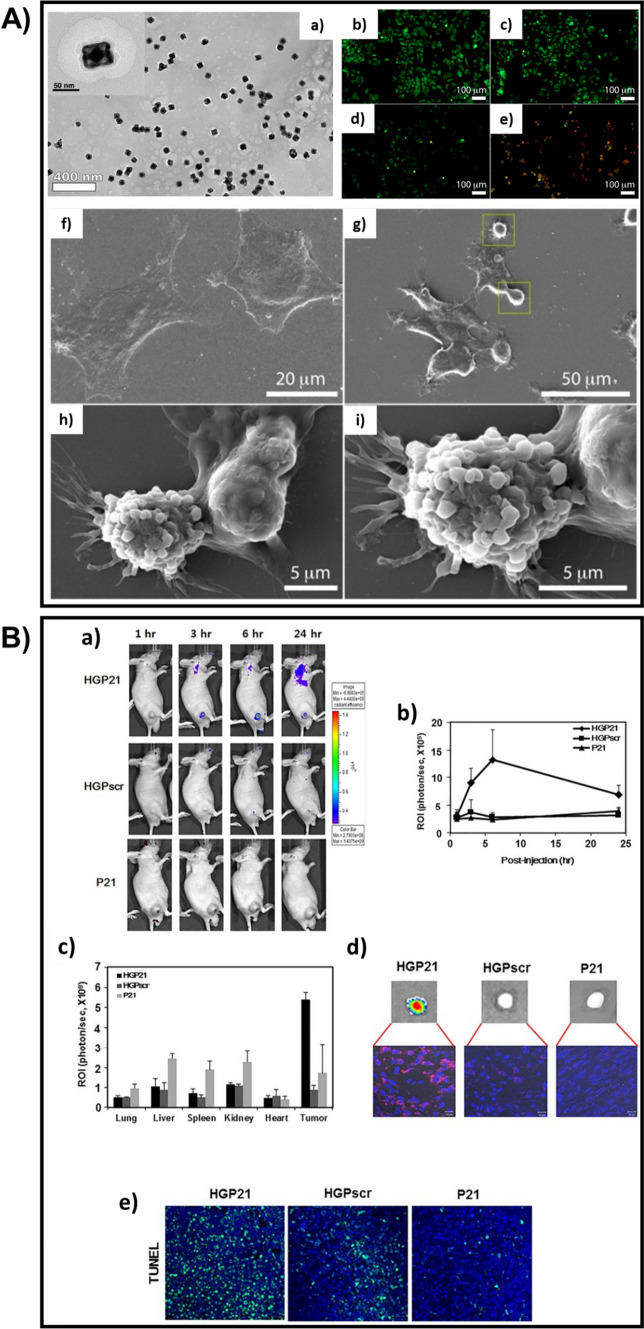
**A** (a) TEM image of Au nanorattles enclosed within chitosan. (b-e) Fluorescence microscopy images of Acridine orange/ethidium bromide dual staining of MCF-7 cells treated with 200 μgml^−1^ of the nanocarriers under different conditions: (b) control, (c) CS-Au nanorattles-DOX without laser, (d) CS-Au nanorattles-DOX with NIR laser. (f-i) SEM images of MCF-7 cell apoptosis: (f) untreated; (g-i) MCF-7 cells treated with 200 μgml^−1^ CS-Au nanorattles-DOX irradiated with 785 nm NIR laser. Adapted with permission from [118]. Copyright 2019 American Chemical Society. **B** (a) In vivo fluorescence images of MDA-MB-231 tumor-bearing mice captured at 1 h, 3 h, 6 h, and 24 h post-injection of Cy3-labeled HGP21, HGPscr or P21. (b) Fluorescence intensity of each tumor site by region of interest analysis. (c) Fluorescence intensity of isolated organs from each group at 24 h post-injection. (d) Ex vivo fluorescence images of isolated tumors, and their tissue sections using confocal microscopy (e) Observation of apoptotic activity in each tumor section of nanoparticles-injected mouse, by TUNEL assay. Adapted with permission from [186]. © 2017 Elsevier Ltd. All rights reserved

An NIR-responsive, non-toxic nano-drug delivery system, in which Au nanorattles along with Doxorubicin and a phase-change material were enclosed within chitosan (Fig. 3 A (a)), was developed by Sarkar et al. The Au nanorattles have exceptional characteristics like its structure, which is shell-like and porous, and the electromagnetic hotspots, which are inherent. The developed nanocarrier was found to be receptive to NIR radiations since Au nanorattles displayed a broad spectrum of absorption in the NIR range. It then melted the phase-change material photothermally and resulted in the instantaneous release of Doxorubicin. The NIR-irradiation and the release of the drug significantly inhibited the MCF7 cell lines (Fig. 3A(b–i)) [118]. Zhang et al. developed a biocompatible, nanoparticle-based probe that can be used for both photothermal and photodynamic therapies. The shell of the nanosystem contained mSiO_2_ loaded with Chlorin e6 (a photosensitizer-Ce6) and modified by catalase, the core was made of gold nanostars (AuNSs) and Cyclo (Arg-Gly-Asp-D-Tyr-Lys) was used to enclose the nanosystem and also as the targeting agent. AuNSs are highly efficient in thermal conversion and thus the approach of utilizing them as the core increases the effect of photothermal therapy. Also, the modification of the shell using the catalase helped in avoiding the occurrence of hypoxia, as the catalase converts the H_2_O_2_ formed in the tumour cells into oxygen. The nanoprobe was found to be capable of selectively recognizing the tumour cells, releasing Ce6 to the tumour site alone when triggered, and achieved substantial cytotoxicity on the tumour cells [187].

Hwang et al. developed a theranostic nanosheet using graphene oxide conjugated with hyaluronic acid, loaded with PNA-21 (antisense), and labelled by cyanine-3, to overcome the problems while administering the anticancer drug in-vivo, for example, the ambiguity in the reactions of the therapeutic drug delivered. The nanosystem was reported to be proficient in detecting and impeding the microRNA-21 which supports the growth of malicious tumours. And also, an intense signal of fluorescence was retrieved, 3 h after the delivery of the nanosheet in-vivo (Fig. 3B). Thus, the nanosheet could be efficiently used as a theranostic nanotool and also for delivering the therapeutic nucleic acids [186]. Wu et al. reported the development of a biocompatible and stable phototheranostic nanosystem made of graphene oxide (GO), gold nanostars (AuNSs), polyethylene glycol, and Chlorin e6. The composite exhibited excellent absorbance in the NIR region due to the presence of AuNSs and GO, and therefore enhanced efficacy in the photothermal conversion. It also entirely eradicated the cancerous EMT6 cell lines. So, by ablating using single-wavelength radiation, the developed theranostic tool could be used for synchronized photothermal and photodynamic therapies, and also for effectual photothermal imaging [188]. Diaz-diestra et al. developed a nanoparticle composite for delivering drugs, which can vanquish the concerns regarding the existing nano-based theranostic tools, for example, the toxicity, its reduced ability to disperse in biological fluids, and its re-accumulation over a period. The nanocomposite was made of a combination of rGO and Mn-doped ZnS quantum dots, then modified using folic acid and loaded with DOX. Due to the adsorption of DOX onto the rGO surface through $\uppi$-$\uppi$ stacking and because of the bonding between the loaded drug and the metal ions on the surface (Zn^2+^), the nanocomposite exhibited better loading and entrapping efficacies. The functionalization done using folic acid has helped to reduce the toxicity and also the attraction between the edges of rGO and the walls of the cell membranes, which supported increased dispersion and reduced agglomeration. The presence of folic acid also increases the capability of the nanocomposite to specifically target the tumour cells that are rich in Folate [189]. Kumar et al. developed a stable, hybrid nanosystem by electrostatically assembling GO (2D) and graphene QDs (0D) in layers, using PEI as a bridge. Even when exposed to a laser of very low power, the complex system responded exceptionally, concerning the photodynamic and photothermal activity, bioimaging, and oxidative stress in the MDA-MB-231 cell lines [190]. Luo et al. synthesized GO nanosheets loaded with very small SPIONs of diameter less than 5 nm, with the help of Na_3_C_6_H_5_O_7_ as an inhibitor for crystal growth, which could be utilized for both chemotherapeutics and T1-MRI. Doxorubicin was then modified with generation 2 PAMAM (dendrimer) and cis-aconitic acid since the amino groups can easily form appropriate links with the –COOH groups present on the surface of GO, and this modified DOX was loaded onto the synthesized SPIONs-loaded GO nanosheet. The nanosheet could be used as an efficient drug carrier as it displayed a manageable pH-sensitive release of DOX and better anti-cancer efficiency. Due to the interface formed between the in situ-grown SPIONs and GO, the nanocarrier showed high values of r1 and therefore, excellent performance in the case of T1-MRI in-vivo [191]. Usman et al. also developed a GO-based theranostic drug delivery system in which a natural anticancer drug, Protocatechuic acid, and a contrast medium, GdH_12_N_3_O_15_, were doped onto the GO nanosheet surface through pi stacking and H-bond formations. Later, via electrostatic interactions, gold nanoparticles were adsorbed onto the functionalized nanosheet. The release of the drug was reportedly higher in an acidic environment, and the nanocarrier was non-toxic to normal fibroblasts. A visible increase in the T1-contrast of the developed nanosheet suggested that they could be used as efficient agents for MRI [192]. Further research has to be done here since the results are completely based on the experiments done in-vitro. Guo et al. developed a uniform-sized nanoplatform for the combined photothermal chemotherapy. The nanosystem consisted of rGO onto which mSiO_2_ was grown through the formation of a supramolecular interface, and PEG-modified-Octadecanoic acid was added in order to make the nanoplatform more stable and soluble. Finally, via non-covalent bonding, DOX was loaded to the nanocarrier system. It was reported that the DOX release was initiated only by an acidic medium and when subjected to light radiation, the nanoplatform exhibited improved as well as combined photothermal and chemotherapeutic effects [193]. Another work based on GO was done by Liu et al. in which, GO was PEGylated and then loaded with a 2-photon compound (BL4) and photosensitizer (PPa) simultaneously, for the combined photothermal and photodynamic therapies. The 2-photon compound was found to be capable of converting the incoming light in the NIR region (980 nm) to the light in the visible region and therefore attained a greater, extended therapeutic efficacy. GO quenches the photoactivity of the 2-photon compound and the photosensitizer, but as soon as they are released from the nanocarrier, they are activated. So, the combined therapy with an excitation laser of a single wavelength repressed the large growth of tumour cells and also resulted in lesser damage to the normal cells [194].

The role played by the AgNPs in the field of cancer theranostics is also inevitable. In the work by Debnath et al., AgNPs, whose size could be tuned, were synthesized using the one-step process of vibration milling at high speeds, and in order to reduce the synthesized AgNPs, chitosan, PEG and PVP were utilized instead of surfactants. The particles were found to be stable for a long period and its standard diameters were in the region between 3.1 ± 1.4 nm and 22.8 ± 5.8 nm. They exhibited high levels of cytotoxicity and anticancer characteristics by subduing the growth of MCF-7, NIH-3T3, and NCI-H358 cell lines [195]. Asha et al. developed a nanoparticulate system in which the surface of nanorods of Eu doped HAp was decorated with Ag^2+^ ions that were passivated with linoleic acid. When annealed at 250ºC, via diffusion, ultra-small AgNPs were formed on the surface of nanorods by nucleation. These biocompatible nanorods exhibited outstanding cytotoxicity against MCF-7 and F929 cancer cell lines. The presence of Ag^2+^ ions and AgNPs additionally to europium, made the nanorods display varied luminescence i.e., from NIR to visible range emissions, which makes the developed nanosystem ideal for imaging in the deeper areas of tissues [196]. Yao et al. developed an antibody therapeutic system by conjoining AgNPs with Rituxan, which is a well-known monoclonal antibody for lymphoma. This conjoining with AgNPs restricted the entry of Rituxan into the cells and extended the interaction between the cell and drug, which resulted in enhanced therapeutic efficacy. Thus, the designed nanocarrier has transformed the actions of the antibody at the molecular level. They could also be functioned as a sensitive probe, to identify lymphoma cells that are alive, with the help of SERS. In order to increase the efficiency further, the structural features of the nanocarriers can be altered or the amount of antibody involved can be managed [197]. Sakr et al. also prepared 21 nm hydrodynamic sized, PEG capped AgNPs doped with ^131^I (core–shell), by a unique single-step method. The synthesized particles were found to be highly stable (in-vivo and in-vitro), non-cytotoxic to WI-38 normal cell lines at lower concentrations, enhanced yields from radiolabelling, and improved uptake of tumours. Thus, these particles could be potentially used as nanocarriers for radiotherapeutics in cancer theranostics [198]. Of all the AgNPs shapes, it was conveyed that the Ag nanoprisms exhibit very strong SPR in the NIR region and hence they have greater potentiality for photothermal therapies. But the complication lies in its heavy toxicity and its vulnerability in the physicochemical atmospheres. This inspired Zeng et al. to design a hybrid nanosystem with polydopamine-coated Ag nanoprisms, functionalized with RGD peptide. Polydopamine helps the nanoprisms to stay stable and biocompatible in-vivo, to convert incoming light to heat upon NIR irradiation, and also make the surface ideal for functionalization. So, in short, the developed nanosystem can act as drug delivery system, probe for photothermal therapy and an agent for imaging simultaneously [199].

In the work by Feng et al., SPIONs along with PEG were utilized to encapsulate the mesoporous and hollow copper (II) sulphide nanoparticles, loaded with Doxorubicin. It was noted that the developed nanocarrier system could be effectively controlled with the help of an external magnetic field and also exhibited higher absorption when irradiated using NIR radiation. The efficient encapsulation by the SPIONs resulted in minimal adversities and timely and controlled release of doxorubicin during the in-vivo delivery of drugs. Therefore, the developed system can be used for both photodynamic and photothermal therapies at the same time due to the combined effect of hyperthermia and plasmonic resonance [200]. Hayashi et al. developed a biocompatible nanosystem in a core–shell arrangement, in which the core was formed by a cluster of IONPs. In the cluster formed, the distance between the particles was maintained to be zero and this helped in boosting the r2 value and also the power for generating heat. The process of release of drugs was initiated as a response to the varying magnetic field and even when the magnetic field was removed, the release continued. This resulted in the accumulation of nanoparticles in the tumour-site which in turn facilitated the conception of MRI. So, the consequent application of a magnetic field could generate hyperthermia and restricted tumour cell growth. This led to increased therapeutic efficiency with minimum side-effects [201]. As discussed earlier, the presence of IONPs as a core permits its application in imaging and at the same time, the property of AuNPs (as the shell) getting heated when subjected to laser radiations, the action of attachment of drugs onto its surface is inverted. These properties were exploited by Malekigorji et al. and then synthesized a hybrid nanosystem using IONPs and AuNPs, onto which the drug (which is in the bisnaphthalamido-based series) was loaded. A drug release which was activated by heat was attained as a result of the utilization of the electrostatic interaction between the drug and the Au surface [202]. Huang et al. developed a nanoparticle-based system from PEI and PEG-coated SPIONs, modified with folic acid, and then loaded with Doxorubicin (DOX). The DOX release was highest in acidic medium and the nanosystem exhibited outstanding stability. Also, they inhibited the growth of cancer cells with improved efficacy, in the presence of the magnetic field. A higher value of r2 was shown while monitoring the accumulation of the nanoparticles around the tumour cells, using MRI [203]. Aeineh et al. also developed a delivery system by functionalizing spherical IONP-surface with Glutathione and PEI for delivering Curcumin. The nanosystem exhibited excellent biocompatibility and increased cellular uptake [204]. Gao et al. developed a similar biocompatible nanocarrier using IONPs that were functionalized using folic acid, PLGA, a cell-penetrable peptide, and Doxorubicin. Due to the presence of the peptide, the level of toxicity was significantly reduced and was also having the exceptional ability for tumour-specific targeting. This nanosystem was also capable of encouraging ROS production which resulted in cell apoptosis. They could also be utilized as a probe for MRI r2* mapping [205].

Efremova et al. synthesized a hybrid nanomaterial comprising of AuNPs and Fe_3_O_4_ of selective sizes. When nanoparticles of various sizes were used, ranging from 3 to 11 nm for AuNPs and 6 to 44 nm for Fe_3_O_4_, the volume ratio of Au to Fe_3_O_4_ was maintained constant. Out of various samples, those with 25 nm size exhibited the finest properties that are required for local magnetic hyperthermia and also for being acting as a contrast agent for MRI. The properties included good values for specific loss power and relaxivity of r2, and also excellent efficiency in killing the breast cancer cells in the presence of a varying magnetic field [206]. Beeran et al. developed a theranostic system by embedding SPIONs in Hydroxyapatite nanoparticles, for enhancing the negative contrast in magnetic resonance imaging and also for site-specific hyperthermia. HeLa cell destruction was confirmed by ESEM and cell death of about 75% of the total cancer cell population was observed when they were exposed to low concentrations of the synthesized material (2 mg/ml) for 30 min under a magnetic field (33.8 mT) [207]. Another approach in fabricating a theranostic system using SPIONs that is capable of delivering drugs to tumour cells and performing MRI was done by Xie et al., where SPIONs modified with oleylamine, chitosan modified using PEG and Doxorubicin (DOX) were co-precipitated together and was formed into small cluster bombs. A reduction in the diameter of the synthesized cluster bombs was noted when compared to that of the DOX loaded PEG-modified chitosan particles, which could be attributed to the occupancy of oleylamine-modified SPIONs. The clusters were capable of de-assembling and re-self-assembling with an enhanced saturation magnetization, in the course of in-vitro drug release which was activated by variation in pH. The accumulation of DOX in the nuclei of cancer cells was seen from the results of confocal microscopy and this proved the effectual on-demand anti-cancer drug delivery [208]. The accomplishment of even heating of the cancer cells at a preferred temperature by the agents and the significant valuation of the extent of distribution of the delivered agents are some of the glitches in the area of magnetic hyperthermia mediated cancer therapy. Du et al. designed CREKA conjugated IONPs to overcome such challenges. CREKA, a peptide that targets tumour, was conjugated to improve the distribution over the affected area. Due to the presence of IONPs, the MPI and MRI signals were significantly enhanced and more efficient ablation of the tumour was attained [209]. Lin et al. developed a nanoplatform from polydopamine and Fe_3_O_4_, coated with Hyaluronic acid, and loaded with Doxorubicin (DOX) with the help of a disulphide linker, for combinational chemo and photothermal therapy including thermal and magnetic imaging. The release of DOX was found to be activated under NIR irradiation and also in the presence of glutathione and suitable pH. From both the in-vivo and in-vitro evaluations, much lower cell viability (16.2%), good T2-MR imaging contrast, and enhanced anti-tumour efficiency were reported due to the combined effect of photothermal and chemotherapy. The synthesized nanosystem showed higher uptake by the CD44^+^ HeLa cells than the normal cells which portray the selectivity strategy of the as-synthesized material and exhibited good biocompatibility due to the presence of Hyaluronic acid [210].

In some cases, photosensitizers and nanoparticles are conjugated to form a composite, and when assisted by X-Rays, certain optical characteristics of the composite are enhanced. Therefore, these types of nanocomposites along with X-Ray radiations could be made use for imaging and theranostics of deeper-seated tumours. One such research was done by Jain et al. in which a nanocarrier system was developed using Gadolinium aluminum nanoparticles doped with cerium nanoparticles, then coated with mSiO_2_, and later the Rose Bengal was loaded onto it. When the nanocomposite was irradiated using X-Rays of energy 55 kV, it generated high amounts of singlet oxygen, in comparison with Rose Bengal alone. The nanosystem ominously inhibited the growth of MDA-MB-231 cancer cells, when irradiated using the light of 470 nm wavelength, and also it exhibited efficient magnetic susceptibility. So, the magnetic characteristics and effective photoluminescence of the nanoparticle system makes them applicable for magnetic photodynamic therapies and subjugates the present-day limitations in theranostics, like the restricted depth of permeation of light [211]. dos Apostolos et al. reported the preparation of P(MAA) functionalized Eu-doped SiO_2_/HAp nanocomposite for theranostic applications. The utilization of europium as dopant effectively resulted in the photoluminescent potential of the nanocomposite. Also, the functionalization using P(MAA) enabled pH dependent release of the loaded drug [212]. Kermanian et al. reported the hydrothermal synthesis of IONPs-HAp nanorods, where CTAB was used as the surfactant. In order to analyze the pH-sensitive drug release and the corresponding cellular uptake, curcumin was loaded onto the synthesized nanorods (Cur@IO-HAp). Cur@IO-HAp exhibited better cellular uptake in comparison to the free curcumin. Also, IONPs-HAp nanorods were found to be non-toxic, excellent pH-responsive drug vehicle as well as a promising MRI T2 contrasting agent ideal for cancer theranostics [213]. Mushtaq et al. fabricated spherically-shaped Mn_3_O_4_-HAp nanocomposite with particle size $\sim$ 28 nm. The functionalization of the nanocomposite by the Pluronic® F-127 copolymer as well as folic acid (FA) resulted in the enhancement of drug loading ability, biocompatibility as well as the targeting accuracy. Additionally, the nanocomposite portrayed a promising T1-Magnetic resonance imaging with r_1_ relaxivity of 2.166 mM^−1^ s^−1^, attributing to the presence of Mn_3_O_4_. Also, under UV radiation of lower intensity, the metformin-loaded nanocomposite demonstrated excellent cellular uptake along with good ROS generation, which substantiated their excellent photodynamic therapeutic efficacy [214].

#### Drug delivery across blood–brain-barrier (BBB)

**Fig. 4:**
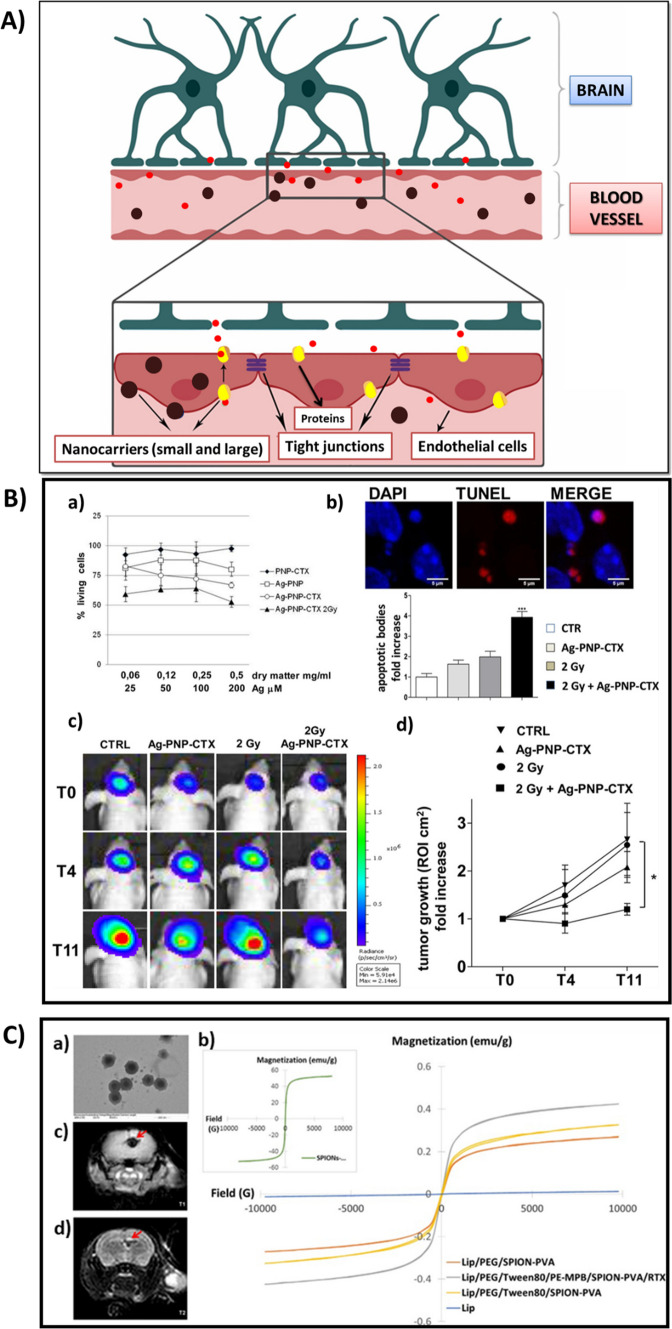
**A** The transportation of large and small nanocarriers across the BBB. **B** Effect of the AgNPs entrapped PNP-CTX on both the irradiated and non-irradiated U87MG cells and the tumor viability. (A) MTT assay of varying concentrations of nanoconstructs for 72 h. (B) In situ apoptosis detection and the corresponding quantification of the apoptotic bodies in U87MG cryosections from the non-irradiated and irradiated mice, injected with AgNPs entrapped PNP-CTX. **C** Luciferase imaging of the representative mice. **D** Plot representing the tumor growth during 11 days of observation. Adapted with permission from [222]. Copyright 2016 American Chemical Society. **C** (a) TEM image of SPIONs-PVA loaded liposome (40,000X). (b) Graph explaining the superparamagnetic properties of the prepared SPIONs-PVA, liposome and SPIONs-PVA-loaded liposome. (c, d) SPIONs accumulation detection by T1 and T2-weight MRI in mice bearing intracranial lymphoma xenografts. Adapted with permission from [223]. Copyright 2017 American Chemical Society

The brain is an ultra-sensitive and delicate system which requires a steady supply of nutrients and other necessities regularly, in order to sustain its functions. The Blood–Brain Barrier or BBB is an arrangement of blood vessels, in a particular manner, for the central nervous system (Fig. 4 A). BBB prevents the delivery of most of the drugs to the central nervous system. Thus, BBB is considered a very strong barrier. The blood–brain barrier is mainly made of the BCECs or brain capillary endothelial cells. It is also composed of various other types of cells like the neuronal cells and pericytes. There are tight junctions present in between the brain capillary endothelial cells. These tight junctions are continuous and they avert or restrict the transport of substances across the epithelium between the brain and blood which results in high transendothelial electrical resistance (TEER). There are various diseases like Parkinson’s disease, Alzheimer’s disease, and tumours that adversely affect the CNS. Since there is restriction by BBB, the appropriate treatment for these diseases is really far from being remarkable. So, for efficient transport of drug carriers, various strategies are to be essentially developed using nanoparticles of very small size, so that these restrictions can be surmounted.

The antibodies that are made by duplicating the immune cells are the monoclonal antibodies (moAb). Cabezón et al. had worked on 8D3 antibody-coated AuNPs and successfully illustrated the process of individual internalization of AuNPs in the vesicles which were mediated by the transferrin receptors. The uptake of the particles by the cells and intracellular drug transporting mechanisms were understood, in detail, using TEM (2D) and SBF-SEM (serial block-face scanning electron microscopy) (3D). The 3D analysis using SBF-SEM, made the researchers understand that those vesicles containing AuNPs are suggestively bigger and more intricate than defined in the 2D study. The author suggested that his work could critically contribute to the improvement of drug carriers that can travel across the BBB smoothly [215, 216]. In the cases of targeting the tumours, especially in the brain, the particle size is considered to be a major factor in the designing of carriers for the drug. Feng et al. designed dynamic switching enabled-gold nanospheres of size ~ 80 nm. These nanospheres exhibited high penetrability, reduced toxicity, and also pH-dependent drug release [217]. By implementing the idea of imaging to the conventional drug carriers, it will be easy to monitor the path of the carrier and also the efficacy of drugs at the targeted sites. One such nanoplatform was developed by Tomitaka et al. by combining liposomes, magnetic nanoparticles, and AuNPs. The drug, Tenofovir disoproxil fumarate, which is used for the treatment of HIV-1, was also incorporated into the nanoplatform. Due to the presence of AuNPs, the drug carrier system displayed increased sensitivity when undergoing CT. And because of the magnetic nanoparticles, targeting the tumour cells became easier and showed improved penetration of BBB, in vitro [218]. Johnsen et al. has investigated the uptake of antibody-coated-AuNPs targeted towards the transferrin receptors present on the surface of the brain capillary surface. It was found that the cellular uptake strongly depended upon the valency and attraction of the antibody-coated AuNPs, and antibodies that are monovalent had improved AuNP uptake efficacy. Also, antibodies with less attraction exhibited a temporary acceptance of AuNPs to the brain and at the same time, those with a high level of attraction mediated a lower uptake [219]. Kang et al. has studied the penetration efficiency of various sizes of the AuNPs through BBB, for curing brain tumours. Gold nanoparticles of 10 nm, 50 nm, and 100 nm sizes were labelled with fluorescent particles and their effects were studied on a mouse with glioblastoma. It was found that the AuNPs of size 10 nm were the only ones that could enter through BBB and distribute over the tumour cells when compared to 50 nm and 100 nm-sized AuNPs that could not enter the brain. The particles were dispersed on the tumour cells alone and did not affect the normal cells [220]. Another work, for improving the ability to deliver drugs for glioblastoma across the BBB, was done by Coluccia et al. 7 nm-sized spherical AuNPs functionalized with an uptake peptide and Cisplatin was investigated in order to understand their potential in treating the tumour cells and enhanced uptake by cells and inhibition of cell growth, which depended on the dosage, was revealed. The treatment was supported by magnetic resonance-guided focussed ultrasound to strengthen the effect of developed nanoparticles [221].

The work done by Chen et al. gives awareness on the level of toxicity of silver nanoparticles when they are used for transportation across BBB. For the study, AgNPs of 8 nm size were used and it was observed that both the silver ions and silver nanoparticles were able to lessen the TEER value so that they could create a discontinuity in the tight continuous junctions of BBB. When the AgNPs and lipopolysaccharide together were used for treatment, an enhanced permeation was reported. But at the same time, the penetrating ability of the nanoparticles was much higher when compared to that of the Ag^+^ ions. Thus, it was said that the intensities of toxicity of Ag^+^ and AgNPs differ. It was also noted that, for evaluating the toxicity effects of AgNPs, in addition to their size, the effects specific to particles should also be considered [224]. Tamborini et al. and the group examined the effects when the nanoparticles are used along with radiotherapy for circumventing BBB. For the studies, they used PLGA nanoparticles (PNP) linked to the peptide Chlorotoxin (CTX), and AgNPs were trapped inside these nanoparticles. The exterior part of the tumour contains more number of attacking cells, rather than the interior part. It was found that the tumour cells at the periphery were much easier to access, by the nanoparticles under study, assisted by radiation. The role of AgNPs comes here as they help in demonstrating the distribution of the nanoparticles over the tumour site periphery. The incorporation of AgNPs aided the nanoparticle system in being a worthy tool for precise imaging, with improved quality (Fig. 4B) [222].

Dong et al. has tried to combine an innovative drug carrier with targeting chemo and photothermal therapies for curing malicious glioma. The drug delivery system was composed of polyethylene glycolated nano graphene oxide conjugated with transferrin and was loaded with Doxorubicin. It was noted that the synthesized nanocarrier had longer endurance during the combinational therapy when compared to that of the non-targeting carrier or the solo drug. Also, the combinational therapy along with the drug carrier could easily cross the BBB and caused an increased cancer cell death rate. Thus, it can be said that this drug delivery system is found potent for travelling across BBB and in treating glioma [225]. Su et al. has done work on the comparison of the permeation ability of two-dimensional GO sheet and GO sheet conjugated with hydrophobic Porphyrin across BBB and it was examined using an in-vitro model. It was realized that the surface-modified GO sheets attained higher efficiency in permeation, in comparison with pure GO sheets. Moreover, Porphyrin modified GO sheets were centrifuged at different velocities in order to obtain particles of varied sizes. The supernatant contained smaller particles and the sediments contained larger particles out of which, larger-sized particles had sharper edges. The improved capability of larger particles for permeation through BBB can be attributed to the contact between the cell membrane and their sharp edges. This penetration was found non-toxic because the Porphyrin modified GO sheets not only penetrated the cellular membranes but also the organelles. Thus, the functionalized GO sheets could be utilized for transporting specific drugs for brain diseases due to their promising permeation ability [226]. Huang et al. was quite successful in developing an efficient drug carrier and combining it with hyperthermia-based chemotherapy, for treating tumours found in the remote areas of the brain. The nanoparticulate system was co-assembled with SPIONs coated with oleic acid, PLGA, poly (γ-glutamic acid-co-distearyl γ-glutamate), and the drug Paclitaxel (PTX). This nanoparticle system was then enclosed within the adipose-derived stem cells (ADSCs) and was therefore found non-toxic to cells even at a higher concentration (30 $\upmu$M) of PTX for 48 h without any stimulus. In the presence of a strong magnetic field, the carrier system exhibited a much higher (four-fold) therapeutic efficacy when compared with conventional chemotherapy using Temozolomide [227].

For the upcoming progressions in treating psychiatric and neurological diseases, there is a need for neuropeptides with targeting capability. But the difficulty here is the inability of such peptides to capably crossing the BBB. ASV-30, from earlier studies, is one such peptide which helps in reducing typical anxiety behaviours by acting upon the CRF2 expressing neurons when used directly. Vinzant et al. verified whether the iron oxide nanoparticles conjugated with ASV-30 could travel across BBB, localizes the CRF2 receptors, and could reduce anxiety. They were successful in their research since there was an enhancement in the bioavailability of ASV-30 by the thorough administration of the iron oxide-peptide (ASV-30) complex nanoparticles. This study helped in introducing a new method for the delivery of peptides, across BBB, with high efficiency and biodistribution [228]. Saesoo et al. developed SPIONs functionalized with liposomes (Fig. 4C(a)), the drug Rituximab and tween80 (for improved penetration of BBB), which was aimed to easily travel across BBB and treat CNS lymphoma. The drug carrier system exhibited higher monitoring abilities, improved delivery of drugs, and was also capable of diagnosis. Also, the spherical-shaped particles of diameter between 140 and 190 nm, showed the property of super-paramagnetism (Fig. 4C(b)). Thus, the developed system could be used as a theranostic tool, which could easily transit across BBB and focus around the cancer cells (Fig. 4C (c, d)). This was one of the first nanoparticle-based theranostic systems ever developed for treating CNS lymphoma [223].

Another temperature-sensitive, Doxorubicin encapsulated liposomal nanosystem was designed by Shi et al., which is capable of crossing BBB and delivers drugs for treating aggressive tumours like GBM. The drug, doxorubicin, and SPIONs were loaded up simultaneously to a liposomal system containing Tenascin-C (antibody) and P1NS (a peptide that is capable of targeting GBM and has high permeability through cells). The results proved that the liposomal delivery system was not eliminated by the endothelial cells and at the same time was capable of targeting and entering the glioblastoma cells. The investigation on the passage of the synthesized delivery system through BBB was done in an in-vitro model and prominent outcomes, like the noticeable value of TEER and expressions of tight junctions, was observed. In the presence of a varying magnetic field, the SPIONs inside the delivery system caused a thermally activated release of doxorubicin, which was controlled, effective, and was also less cytotoxic to normal cells [229]. Shen et al. also worked on developing a nanocarrier, which could easily pass through BBB, act as a probe for imaging, and can function as a theranostic tool, for treating glioma. The nanosystem consisted of hydrophobic SPIONs coated with DSPE-PEG2000, Doxorubicin, and a cyanine dye – ICG. The as-synthesized nanoparticle system was of suitable size (22.9 nm diameter), and thus an effectual uptake of doxorubicin by the cells was observed, without any side-effects. The nanoparticles were able to successfully travel across BBB and were also seen gathering around the tumour cells, in particular, which was observed from the MRI and fluorescence imaging [230]. Shaghaghi et al. designed a multifunctional, SPIONs and folic acid incorporated, Janus-based nanocarrier that could deliver doxorubicin across BBB to the tumour cells in the brain. The surface of Janus nanoparticles is said to have more than 2 physical characteristics and allows 2 separate kinds of chemistry to happen simultaneously. The folic acid conjugated covalently to the Janus nanoparticle acts as an agent for targeting the cancer cells. The developed drug carrier system showed an increased circulation period in blood. Since the doxorubicin was conjugated covalently to the system through imine bonds, a pH-sensitive release of doxorubicin was initiated [231]. Next is the work done by Norouzi et al., on the development of a biocompatible, magnetically targeting vehicle for the delivery of Salinomycin to the brain, for the treatment of GBM. Iron oxide nanoparticles were combined with PEG and PEI and then the drug was loaded onto this system. The release of the drug from the delivery system was prolonged for about four days, in acidic conditions, and was found to be efficient in killing the ROS (reactive oxygen species) mediated GBM cells. The BBB permeability of the Salinomycin-PEI-PEG-Iron Oxide nanoparticle-based system was only about 1.0% ± 0.08%, but in the presence of an external magnetic field and also with hyperosmotic disruption, the permeation ability increased to 3.2% ± 0.1% [232].

When a stroke happens, an excess amount of ROS is produced and they could destroy the BBB. As far as Nanoceria is concerned, they are found efficient in scavenging ROS and offer high protection to the neurons and vasculature of the brain. Bao et al. designed and developed a neuroprotective nanocarrier to effectively deliver the therapeutics required for the treatment of strokes. The nanosystem consisted of CeO_2_ nanoparticles functionalized with PEG and an oligopeptide, Angiopep-2, and was loaded with the drug, Edaravone. This system was non-toxic, was able to cross BBB through transcytosis, and at the same time, was successful in ensuring the stability of the environment of the brain. A high level of agglomeration of particles was found in the intracerebral lesions. This nanoparticle system could, therefore, be used for the targeted delivery of drugs for treating neurodegenerative diseases [233]. Kaushik et al. computationally analyzed the effectiveness of ceria nanoparticle-based drug delivery systems in elucidating the activity of α-synuclein (causing Parkinson’s disease), when compared to that of the SPIONs and AuNPs. It was found that CeO_2_ nanoparticles were capable enough to inhibit α-synuclein activity [234]. In a recent attempt by Lin et al., honokiol loaded HAp nanoparticles were developed for targeted delivery of the drug, during the post-glioma surgery management. It was noted that in acidic environment, the hydrophobic honokiol-HAp nanoparticles undergo burst release. The nanoparticle system also displayed better cell viability along with prolonged and effective release of honokiol. Additionally, after the treatment with honokiol loaded HAp, the MRI results of the in-vivo studies carried out, displayed an effective reduction of the size of tumor by 40% [235].

#### Ocular drug delivery

**Fig. 5:**
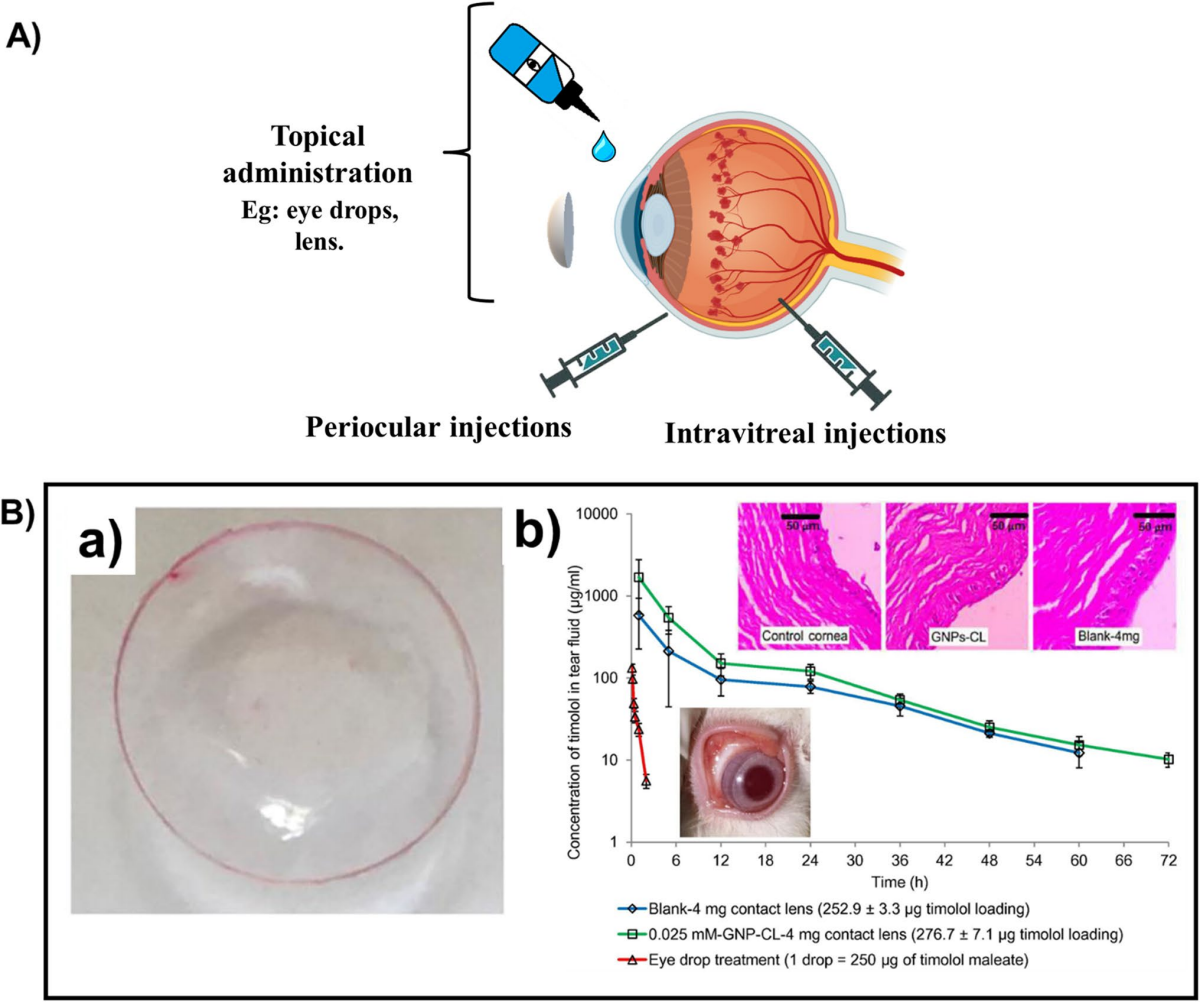
**A** Various methods of administration of drug-loaded nanocarriers to overcome different ocular barriers **B** (a) Image of the AuNPs loaded contact lens. (b) Timolol concentration in tear fluid from the blank-4 mg contact lenses (253 ± 3 μg timolol loading), 0.025 mM-GNP-CL-4 mg (277 ± 7 μg timolol loading) and eye drop treatment (1 drop = 250 μg of timolol maleate). The histopathological images of cornea were taken by the light microscopy at X450 magnification. Adapted with permission from [239]. © 2019 Elsevier Ltd. All rights reserved

The human eye is a very complicated organ with distinctive physiology and anatomy, and therefore, the designing of an ideal drug carrier for the eye, that targets a certain tissue, has been a greater challenge for the researchers. The interior of the eye consists of three parts: anterior and posterior chambers, and vitreous body. The tissues like cornea, iris, and lens, together form the anterior chamber. The posterior chamber is made of tissues like the choroid and sclera. Different types of vision-affecting diseases, for example, cataract, conjunctivitis, diabetic retinopathy, and glaucoma, pose a threat to the anterior chamber and posterior chamber. In the eye, the retina is separated from the circulation of blood, by the BRB or Blood-Retinal Barrier. Both the interior and exterior BRB cells consist of tight junctions that differ only in their configuration. These tight junctions control the movement of molecules or fluids between the tissues in the retina and the ocular vasculature. It also prevents the entry of very large molecules and various other foreign, unsafe agents into the retina and supports the retention of the micro-environment in the retina [236]. In addition to this barrier, there are various other barriers, both static and dynamic which are unique and intrinsic to ocular anatomy. Most of these barriers generally protect the eye from toxic agents [237]. Due to these obstructions, it is important to develop a drug carrier for treating retinal diseases with appropriate size range, enhanced drug penetration, controlled drug release, and drug targeting capability with minimal side-effects which ensure high therapeutic efficiency and biocompatibility (Fig. 5 A).

In a study by Salem et al., an antifungal drug named Flucytosine was capped by gold nanoparticles and the nanocarrier was developed with the help of liposomes for treating the inflammation of intraocular fluids (endophthalmitis), caused by *Candida albicans*. AuNPs acted as an agent for contrasting, which helps in tracing the drug in the posterior chamber of the eye. Liposomes were formulated in varying ratios of molar weights. The optimum formulation of liposome was obtained, when stearylamine, phosphatidylcholine, Span 60, and cholesterol were taken at a ratio of 0:1:1:1, with a maximum ocular depth of penetration (10.22 ± 0.11 mm) and maximum drug release (7.043 mg/hour). When *Candida albicans* infected cornea of rabbit were treated with the as-prepared nanocarrier, it reported an increase in the healing efficiency, since there was an increase in the zeta potential (positive) of the liposome used. The Computed Tomography (CT) imaging technique revealed the achievement in tracking the AuNPs [238]. For prolonged delivery of drugs to the tissues in the eye, contact lenses could be used. But the problem lies in the fact that the inclusion of drugs onto the lens could damage its significant characteristics. There are various methods implemented for loading drugs to contact lenses like molecular imprinting, the technology of supercritical fluids, and soaking methods. Timolol is a medication that helps to treat glaucoma, which is caused by the increased pressure in the eye. Incorporation of timolol to the lens via the standard method of soaking doesn’t expressively change the substantial characteristics of the lens but there is a possibility for burst release and minimal loading of drugs. Maulvi et al. studied the consequences of loading AuNPs on the contact lens, through the conventional method of soaking, and also about its drug-releasing performance. In the first methodology followed, AuNPs were mixed in the soaking solution that contains timolol (GNP-SS), and in the second, the AuNPs were directly fabricated to the lens (GNP-CL) (Fig. 5 B (a)). The in-vivo studies indicated that GNP-CL contained higher concentrations of timolol. The studies on the distribution of drugs also revealed the major enhancement in the deposition of drugs at the desired sites, when GNP-CL was used (Fig. 5B(b)). So, in a nutshell, the author has reported the ability of AuNPs in enhancing the acceptance of drugs from the soaking solution, without affecting the properties of the lens [239]. AuNPs should be highly stable to be more efficient and also to avoid forming clusters in tissues or cells. Masse et al. reported the novel conditions under which highly stable AuNPs were synthesized. The AuNPs were made highly stable by combining it with ligands of mass that range from 800 to 600 g/mol. The research group has also proven the capability of the synthesized nanoparticles in encapsulating the drug used for treating glaucoma, Bimatoprost, from the experiments done for assessing drug encapsulation. The nanoparticles exhibited no cytotoxicity in MTT assay. From the results obtained, the author has concluded that the synthesized ultrastable AuNPs can be utilized as potential drug carriers for ocular therapies [240]. Natesan et al. designed a formulation in which Hypocrellin B, along with AgNPs, was loaded on PLGA (HBS-NPs), which aimed at attaining an increased ^1^O_2_ production and can be applied for ocular photodynamic therapy, i.e., for treatments in the posterior chamber of the eye. The HBS-NPs were in the range between 135.6 and 828.2 nm, showed 92.9 ± 1.79% of encapsulation efficacy, contained 2.60 ± 0.06 mg/mL of amorphous Hypocrellin B and exhibited a negative zeta potential. Regarding the release of drugs, a burst release (3.50%) was witnessed in the initial 8 h, which was then followed by a steady release (47.82%), observed for 3 days. An increase in the ROS production by the HBS-NPs was detected, when compared to that of the HB-NPs or pure HB. When HBS-NPs were irradiated by the light source, it showed phototoxicity which depended on time and concentration [241].

Typically, the RPE cells are held responsible for causing blindness in both adults and children. From the report of Giannaccini et al., effective utilization of magnetic nanoparticles as drug carriers made it possible to rapidly and specifically locate the RPE cells. Additionally, this nanocarrier could be used to offer therapies in those areas which have the least access and can also be used as an agent for tracking in MRI, in various kinds of retinopathy [242]. Mousavikhamene et al. introduced an exceptional method for delivering drugs from the periocular routes, across the sclera, with the help of magnetically subtle nanoparticles that are loaded with drugs. He stated that applying an external magnetic field in front of the eye, after injecting the magnetically-active, polymeric nanoparticles into the periocular space (parallel to the axis of the eye), could influence the nanoparticles so that, they move in the magnetic field direction and travel across the sclera. This method could overcome the difficulties faced by the normal scleral drug nanocarriers. An anti-inflammatory drug, diclofenac sodium, was loaded onto a nanocomposite consisting of sodium alginate (biopolymer) and IONPs. In the presence of an external magnetic field, a substantial upsurge in the transferring of diclofenac sodium through sclera was affirmed [243]. Agban et al. developed a novel nanoparticle cross-linked collagen to overcome the difficulties met while using the usual therapeutics for treating glaucoma. The current practice involves the recurrent usage of eye drops which resulted in reduced healing effects and weak patient compliance. The objective of this research group was a continual delivery of Pilocarpine hydrochloride and surmount the ineffectiveness of the eye drops prescribed for glaucoma. PVP functionalized zinc oxide nanoparticles were selected as ideal delivery candidate when compared with TiO_2_ and pure ZnO. ZnO/PVP loaded with PHCl, displayed cytotoxicity, thickness, tensile strength, transparency in shielding and bioadhesive properties which are supportive for ocular drug delivery. Zinc ions were also released along with PHCl and the concentration of Zn ions was much lower than the half maximal inhibitory concentration. Also, the drug release from the crosslinked collagen was observed for a stretch of 14 days. This confirmed a sustained PHCl release for the therapy, which is much more extended than the drug release by the administered eye drops [244]. Luo et al. has also introduced a new formulation for the treatment of glaucoma. They have designed a nanocarrier (in the form of eye drops) for targeted and continual delivery of pilocarpine to the eye. Hollow nanoceria was functionalized with ZM241385 and chitosan. They were designed in such a manner so that they can pass through the tight junctions of BRB. The results of the prepared eye drops, when compared to that of the conventional eye drops, showed that the as-prepared nanocarrier had anti-inflammatory and antioxidant characteristics, which are the important criteria for eradicating glaucoma progression. It established 42 times longer period to normalize the elevated pressure, in a one-time administration which, according to the author, attributes to the permeation of the nanocarrier through the cornea [245]. Diabetic cataract is considered as one of the major causes of visual impairment in patients, who are diabetic. There are only a handful of drugs that are capable of prolonging or preventing this type of cataract. Zhou et al. synthesized Nanoceria and coated it with a combination of polyethylene glycol and PLGA to form a redox and auto-reformative nanoparticle. The results affirmed that the nanoparticles remained in the eyes for an extended time and thereby reduced the opacity of the lens which, later on, attenuates diabetic cataract. The author suggested that this advantageous result can be attributed to two reasons; the antioxidant behaviour of the prepared nanoparticles and the action of nanoparticles as an inhibitor of non-enzymatic glycosylation which helps to keep the lens transparent [246].

#### Drug delivery for microbial infections and wound healing

**Fig. 6:**
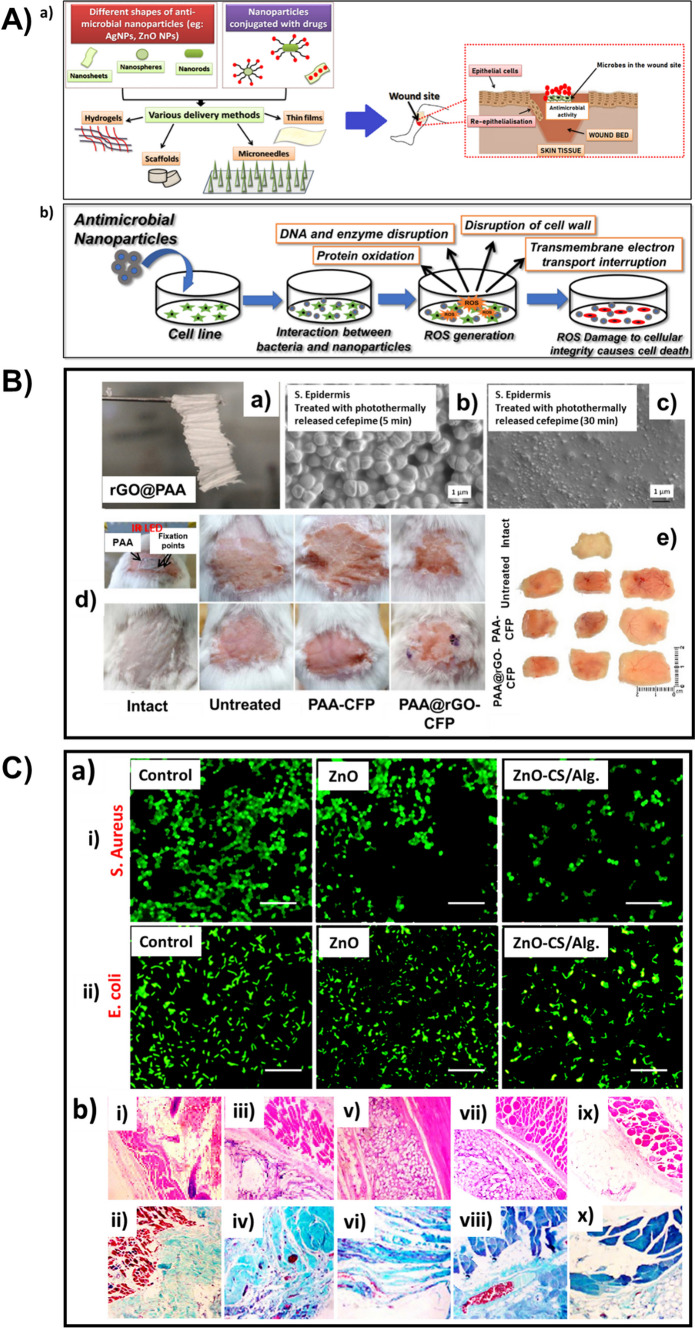
**A** (a) The effect of delivery of naked anti-microbial nanoparticles and drug conjugated nanoparticles to the wound site by various methods. (b) The mechanism of antimicrobial activity of anti-microbial nanoparticles. **B** (a) Photograph of the PAA@rGO electrospun polymeric mat. (b, c) SEM images of *S. epidermidis* treated with cefepime loaded PAA@rGO mats and irradiation for 5 min and 30 min, respectively. Photographs of (d) wound scars, 24 h after photothermal treatment for 10 min and (e) intact mouse skin and the three representative skin samples after 48 h of infection, under different treatment conditions. Adapted with permission from [256]. Copyright 2018 American Chemical Society. **C** (a) The confocal images of (i) S. aureus and (ii) E. coli, respectively treated with ZnO and ZnO-CS/Alg. (b) Histological analysis on HE stains and on Masson’s trichrome stain of the ZnO-CS/Alg. group at (i, vi) 3 days, (ii, vii) 7 days, (iii, viii) 14 days, (iv, ix) 21 days, and (v, x) 48 days, respectively. Adapted with permission from [257]. © 2019 Elsevier Ltd. All rights reserved

The chronic wounds and full-thickness burns are extremely vulnerable to infections caused by bacterial growth and their treatment is quite costly. So, their co-operated healing imposes a huge responsibility for the therapeutics. The top-notch approach for enhanced extermination of bacteria can be made by encapsulating the necessitated drugs into the nanoparticulate system. In this way, the efficiency in delivering the drug to the target site and thus the bioavailability of the drug can be enriched, with reduced toxicity (Fig. 6A(a)). Nanoparticles are known to have a large surface area which makes them easier to functionalize, resulting in furthermore increased ability in loading drugs onto its surface. Therefore, nanoparticles exhibit a very high affinity to bacterial growth (Fig. 6A(b)) [247].

Various biomaterials have been developed recently, that are capable of completely eradicating the bacterial growth and repairing the skin tissues at the wound site, rapidly. AuNPs-incorporated thermosensitive gels were developed by Arafa et al. for the transdermal delivery of AuNPs to act against *S.aureus* growth and heal burn-induced wounds. Two types of gels were developed; Type 1 contained only 15%w/w Pluronic® 127 (a polymer that is thermosensitive) along with AuNPs and Type 2 consisted of both Hypromellose (viscoelastic polymer) and Pluronic® 127 (15:1%w/w) in addition to AuNPs. From the ex-vivo, in-vivo, and in-vitro test results, Type 2 showed better bioadhesion, viscosity, and 100% drug release in 6 h while Type 1 had higher permeation flux and 98.03% release of drug in 6 h. Both Type 1 and 2 had better capability in acting against *S.aureus* and healing the wound and exhibited increased bioavailability when compared to that of the AuNPs in suspension form [248]. Another work using gold nanoparticles was done by Wang et al. in which, AuNPs of about 7 nm were combined with LL37 (Cathelicidin antimicrobial peptide) and vascular endothelial growth factor (VEGF), in order to develop a gene-delivery nanosystem for treating chronic diabetic wounds. The nanosystem showed notably higher capability in inserting the pDNA to keratinocytes, in the outermost layer of skin. It also exhibited high efficiency in antibacterial activity with lesser toxicity levels, endorsed angiogenesis, and thus helped in increasing the rate of closure of the wound [249].

Rangasamy et al. developed a special kind of AgNPs that were conjugated with Pyridoxine, having antibacterial characteristics, moisturizing properties, and properties for healing a wound. It was found that the synthesized nanoparticles were able to fasten the movement of keratinocytes and fibroblasts which supported the therapeutic effect on the wound [250]. Aloe vera is a plant that contains vitamins and amino acids abundantly. It is one of the perpetual elements used in cosmetics and medicines, which also has a soothing effect. These properties of aloe vera were made use by Chabala et al. and combined with the healing and antibacterial characteristics of AgNPs, chitosan-alginate polymer system, for developing a new kind of dressing material for wounds. The polymer matrix with AgNPs, synthesized by the method of blending, was highly porous, which facilitated higher absorption and release of Aloe vera. The matrix in which Aloe vera and AgNPs are incorporated showed antibacterial property towards *P.aeruginosa* and *S.aureus.* This material can be used for wound healing applications in order to reduce the consequences of using antibiotics [251]. Tarusha et al. also developed a blended polymeric membrane consisting of hyaluronic acid, polysaccharides alginate, and Chitlac coated AgNPs, where Ca^2+^ ions were used as an agent for the cross-linking process, for healing chronic wounds. The matrix developed was highly efficient in eradicating and preventing the growth of the biofilms formed by bacteria and planktonic bacteria, and was proven to be non-cytotoxic to normal cell lines, due to the slow-release rate of Ag. The presence of Chitlac coated AgNPs helped in inhibiting the overexpression of proteolysis by Matrix metalloproteinases, which could hinder the wound healing process. The developed membrane possessed a better value of transmission rate of water vapours, which confirmed the presence of moisture on the wound bed, which would not allow any risk of dehydration and helps in the restoration of the tissues [252]. Oryan et al. synthesized AgNPs that were capped with chitosan in a single step and analyzed the outcomes when they were introduced to wounds caused by burns. After 7 days of application of the chitosan-capped AgNPs to the wound, a considerably low inflammation, an increase in the TGF-β1 and bFGF, improved and faster restoration of epithelial cells, and enhanced maturing of granulation tissue was noted. Therefore, it was evident that the chitosan-capped AgNPs were effective and highly encouraged faster healing of wounds caused by burns, by reducing the time taken by each repairing phase [253].

There are some cases where the delivered drugs are ineffective in killing the bacteria at the wound site since they resist the entry of drugs. AgNPs along with the photothermal therapy is being utilized in such situations, but the less effectiveness of the photothermal therapy and the toxicity caused because of the exposure of humans to high volumes of silver, are some of the limitations encountered during the application. To enhance the efficacy of the antibacterial agents, Liu et al. developed a nanocarrier by combining two different bactericidal agents into a single platform. Gold nanorods coated with polydopamine were loaded with Ag^+^ ions and Glycol chitosan which was labelled with Cyanine 5-SE. The developed therapeutic nanoplatform had a higher capacity in loading the Ag^+^ ions, and very low-dose release of Ag^+^ was pH-dependent which led to a selective agglomeration of Ag^+^ ions at the bacterial site. The low dosages of Ag^+^ ions were able to pierce through the membrane of the bacteria and were successfully able to moderate the resistance of the cell membrane posed towards heat, which later on led to the enhancement in the efficacy of photothermal therapy. Also, due to the hyperthermia caused, increased Ag^+^ release was observed, and thus development in the efficiency in killing the bacterial growth. These mechanisms were highly supportive of rapid wound healing and faster recovery from bacterial infection [254]. Chhibber et al. studied the efficiency of a microemulsion made from AgNPs that were coated with an alpha-amino acid, histidine, in healing infections caused by K.pneumoniae at the burn-wound site. There was a significant decrement in bacterial growth and the healing efficiency was improved. The developed microemulsion can be utilized for delivering therapeutics to those sites, where the bacteria which resist the antibiotics are present [255]. Altinbasak et al. developed a stable, nano polymeric mat that releases the antibiotic drugs to the affected area as and when irradiated with NIR radiation, by electrospinning PAA and rGO (Fig. 6B(a)). The mat synthesized displayed precise heating when irradiated with a 980 nm laser. When the antibiotic-loaded mat was exposed to the NIR laser, the rate of release of the drug was enhanced when compared to the rate of release in the absence of the NIR, and the rate was dependent on the power of the laser used. So, the amount of drug released can be controlled externally, based on the required quantity of antibiotics to kill the bacteria (Figs. 6B(b–e)) [256].

Ali et al. explored the capability of rGO in acting against the biofilms, which resist the entry of antibiotics to the wound site and promote chronic wounds. rGO along with C_8_H_15_NaO_8_ (carboxymethylcellulose sodium) formed a hydrogel and was confirmed that the hydrogel was efficient in restraining the growth of biofilms formed by *P. aeruginosa* and *S. aureus*, through the XTT test [258]. Fazli et al. developed a nanofiber-based mat using Chitosan and PEG, onto which Imipenem/Cilastatin-Hydrocortisone-coated ZnO nanoparticles were loaded. The swelling behaviour of the mat was at its maximum in the acidic environment and even after the complete utilization of the drug-loaded mat in buffer solution for a period of 8 days, its antibacterial property was conserved. The rate of release of Imipenem/Cilastatin (reduces infection) was found to be very slow when compared to that of the Hydrocortisone (inhibition of swelling) from the mat, which was appropriate for the wound healing dressings [259]. Gong et al. developed a biofilm from Chitosan, Alginate, and ZnO nanoparticles for managing wound dehiscence, which is quite common in patients who have received sutures in abdominopelvic surgeries. Alginate and chitosan are biodegradable polymers that are well known for their biocompatibility and the incorporated ZnO nanoparticles were observed to have a smaller size and large surface area, which highly influences its antimicrobial activity and its role in wound healing (Fig. 6C(a) (i, ii)). From the images taken after Masson’s trichrome staining, it was seen that the newly formed tissue wall in the wound site in the abdomen, exhibited high mechanical strength with increased deposition of collagen (Figs. 6C(b) (i–x)) [257]. Another work using ZnO nanoparticles was done by Masud et al., for enhanced healing of wounds through the controlled release of antibiotics. ZnO nanoparticles, Chitosan, and PEG were combined and linked through sodium triphosphate, and Gentamicin was loaded (loading efficiency-76%) onto the semi-porous nanocomposite. Because of the combinational effect of ZnO and gentamicin, the nanocomposite displayed increased bactericidal activity towards *S.enterica* and *E.coli*, when analyzed in-vitro. The prepared nanocomposite could be used as an effective wound dressing material since it degraded slowly in both PBS and water, offered moisture at the wound area, and exhibited enhanced healing characteristics by not leaving scars when compared to that of the commercially available hydrogels [260]. Even in the current times, curing an injury without leaving any scars is considered a big challenge due to some complications. Curcumin is well-known for its anti-inflammatory properties in healing any kind of wound. Bhattacharya et al. combined the anti-oxidant properties of nanoceria and the anti-inflammatory properties of Curcumin in order to find whether they could completely heal a wound without leaving scars. A hydrogel scaffold consisting of CeO_2_ NPs, PAA, and Curcumin was developed for dressing an acute wound. The release of Curcumin from the hydrogel was sustained and the efficiency in healing the wound was around 78%. The scaffold was applied only for one time and in a period of 7 days, the presence of renewed follicles of hair and an insignificant scar was found in comparison to that of the dressing material with Curcumin alone [261].

Xu et al. reported a study done on the efficacy of HAp incorporated with AuNPs and coated with polydopamine, in killing bacteria like *S.aureus* and *E.coli*, and healing the wound when combined with photothermal therapy. The free radicals (•OH) produced by the synthesized nanoparticles, make the bacteria susceptible to the change in temperature. At low and regulated temperature (45 °C), the nanoparticulate system was able to kill 95.2% of *S.aureus* and 96.8% of *E.coli*, and initiated skin-tissue regeneration. So, at a lower temperature, the bacteria were killed in a short period with high efficiency without affecting the normal skin tissue [262]. Similarly, Suja et al. prepared Fe-doped HAp as an antibacterial agent for various biomedical applications. Varying concentrations of Fe-doping was carried out in which the 0.2 M Fe doped HAp exhibited prominent antibacterial efficacy [263]. In another attempt by Suja et al., self-luminescent Mg doped HAp was prepared for the roles of antibacterial agent and drug carrier. The analyses were carried out on both the as-prepared as well as microwave irradiated nanoparticles. The microwave irradiated samples exhibited a sustained, quasi-Fickian drug release pattern and thus were found to be ideal for drug delivery applications [264].

## Fate of inorganic nanoparticles post-application

The inherent physical as well as chemical characteristics of the inorganic nanoparticles, and the properties that evolve from these characteristics (such as quantum confinement and superparamagnetic behaviour) could moderately or completely vary as the particles interact with the physiological microenvironment [265]. For instance, a surface modified nanoparticle system when in contact with the physiological environment, could partially lose its coating, which could ultimately lead to instability of the particle system and their aggregation. As the nanoparticles are taken up by the cells, the protein layer protecting them will be enzymatically digested in the phagosomes or lysosomes. After the digestive process in the cell, the degraded nanoparticle system would be considered foreign and this triggers various immune responses inside the host. These degraded particles could initiate certain variations in the physicochemical characteristics of the residue [266]. These variations, like agglomeration of nanoparticles, could alter their biodistribution and also their immunogenicity. Thus, a precise and comprehensive investigation on such variations is prerequisite to clinical trials, especially in the case of inorganic nanoparticles.

## Clinical trials and FDA-approval of inorganic nanoparticles

In the past two decades, the clinical trials and FDA and/or EMA approvals of inorganic nanoparticle-based drug carriers have sky-rocketed. On the basis of 2022 data, more than 100 nanoparticle-based formulations are available in the market [267, 268]. Due to the superparamagnetic behaviour of SPIONs, dextran coated SPION-based FDA approved imaging agents (Feridex®/ Endorem®) are readily available in the market. AuNPs are also one among the few inorganic nanoparticles that are FDA approved. Most of the AuNP-based clinical trials are carried out for applications such as contrast agents, drug delivery and photothermal therapy [269, 270]. Till date, the most accounted FDA approved inorganic nanoparticle is AgNPs, for many of its properties. AgNPs have attained greater commercialization, conquering 57% of the whole commercial products available. Few examples for the biocomposite wound dressing with AgNPs, with the US-FDA approval, are PolyMem Silver™ (Aspen), Aquacel™ (ConvaTec) and Tegaderm™ (3 M) [271]. Thus, conclusively, it is essential to focus on the efficiency as well as safety of the nanoparticles, whilst abiding by the regulations instituted by the agencies like EMA and FDA.

## Outlook

The unification of nanoscience and nanotechnology with biomedicine has opened wide and stimulating paths for research, and some recent developments have indicated the greater potential of the nanoparticles for their applications in drug delivery. This review article has conversed about the physical, chemical, and biological properties of different inorganic nanoparticles, how these properties vary depending on the structure, shape, and size of the nanoparticle considered and has also outlined the exceptional properties of the inorganic nanoparticles which make them useful for designing ideal drug delivery systems. Furthermore, recent research developments in designing drug carriers using AuNPs, AgNPs, graphene-based nanoparticles, Iron oxide NPs, ZnO NPs, CeO_2_ NPs and nano HAp for different drug delivery applications have also been mentioned. Nevertheless, the authenticity of the inorganic particles, when compared to the organic nanoparticles, has been disputed as they are associated with certain restraining factors, which cause souring pain in the biomedical field.

In order to select the ideal nanoparticle for a drug delivery application, it is necessary to consider its factors like biocompatibility, drug-loading capacity, release kinetics, and targeting capabilities. Moreover, a thorough evaluation of the regulatory and safety aspects of nanoparticles in medical applications should be carried out. Extensive studies are being conducted by the researchers recently for determining the most appropriate nanoparticle for a specific therapeutic purpose.

The selection of nanoparticles for certain drug delivery applications also depends on various other factors like the specific drug being delivered, the proposed target, and the anticipated drug release kinetics. i.e., if targeting cancer cells is crucial, then AuNPs or graphene-based nanoparticles might be suitable due to their targeting capabilities. If antimicrobial properties are the prime concern, AgNPs could be considered. Iron oxide nanoparticles are useful whenever magnetic targeting is needed, and ZnO NPs can be utilized for wound healing. Thus, each of the nanoparticles mentioned in this article has unique properties that can make them suitable for drug delivery applications in different contexts.

The development of efficient nanoparticles, capable of delivering multiple drugs or therapeutic agents simultaneously, will gain prominence. This approach can significantly enhance the treatment efficacy and reduce the extent of resistance. Nanoparticles that respond to specific external stimuli, such as pH, light and/or temperature, will be highly explored. These "smart" nanoparticles can release drugs in response to the microenvironment of the target site, suggestively minimizing the systemic side effects. Developing patient-specific drug delivery systems tailored to an individual's disease profile and genetic makeup, would be an exciting avenue to explore. As the nanoparticles advance towards the clinical applications, regulatory agencies will need to establish guidelines and safety standards that are specific to the nanoparticle-based drug vehicles.

Research in this area will involve meeting regulatory requirements and ensuring the product quality as well as safety. Developing cost-effective, scalable production methods for the nanoparticles will be vital for their widespread adoption in the pharmaceutical arena. Transitioning the nanoparticle-based drug carriers from the laboratories to clinical trials and, eventually, to the market will be of significant focus. Researchers will need to address the clinical challenges, such as pharmacokinetics and the compliance by the patients. These research perspectives demonstrate a broader potential of utilization of nanoparticles as drug carriers and highlight the ongoing efforts to advance this field for better and improved healthcare outcomes. A collaborative effort between scientists, clinicians, regulatory bodies, and industrialists would be essential in driving these innovations toward clinical practice.
